# The Wnt Frizzled Receptor MOM-5 Regulates the UNC-5 Netrin Receptor through Small GTPase-Dependent Signaling to Determine the Polarity of Migrating Cells

**DOI:** 10.1371/journal.pgen.1005446

**Published:** 2015-08-20

**Authors:** Naomi Levy-Strumpf, Meghan Krizus, Hong Zheng, Louise Brown, Joseph G. Culotti

**Affiliations:** 1 Lunenfeld-Tanenbaum Research Institute of Mount Sinai Hospital, Toronto, Ontario, Canada; 2 Department of Molecular Genetics, University of Toronto, Toronto, Ontario, Canada; University of Cambridge, UNITED KINGDOM

## Abstract

Wnt and Netrin signaling regulate diverse essential functions. Using a genetic approach combined with temporal gene expression analysis, we found a regulatory link between the Wnt receptor MOM-5/Frizzled and the UNC-6/Netrin receptor UNC-5. These two receptors play key roles in guiding cell and axon migrations, including the migration of the *C*. *elegans* Distal Tip Cells (DTCs). DTCs migrate post-embryonically in three sequential phases: in the first phase along the Antero-Posterior (A/P) axis, in the second, along the Dorso-Ventral (D/V) axis, and in the third, along the A/P axis. Loss of MOM-5/Frizzled function causes third phase A/P polarity reversals of the migrating DTCs. We show that an over-expression of UNC-5 causes similar DTC A/P polarity reversals and that *unc-5* deficits markedly suppress the A/P polarity reversals caused by mutations in *mom-5/frizzled*. This implicates MOM-5/Frizzled as a negative regulator of *unc-5*. We provide further evidence that small GTPases mediate MOM-5’s regulation of *unc-5* such that one outcome of impaired function of small GTPases like CED-10/Rac and MIG-2/RhoG is an increase in *unc-5* function. The work presented here demonstrates the existence of cross talk between components of the Netrin and Wnt signaling pathways and provides further insights into the way guidance signaling mechanisms are integrated to orchestrate directed cell migration.

## Author Summary

Cells are exposed to a multitude of environmental cues that are often eliciting additive, overlapping, or even conflicting inputs. How the information from multiple extracellular cues is integrated within the cell to generate distinct patterning is largely unknown. Netrin and Wnt signaling pathways are critical to multiple developmental processes and play key roles in normal development, as well as in malignancies. The involvement of these two signaling pathways in establishing cellular polarity is key to their ability to determine organ shape and to regulate cell and axon migration. Here, we reveal a regulatory link between the Wnt Frizzled receptor, MOM-5, and the Netrin receptor UNC-5. We present evidence showing that MOM-5/Frizzled signals through small GTPases to negatively regulate the UNC-5 Netrin receptor. This regulatory link enables the integration of Netrin and Wnt signaling pathways and facilitates their orchestrated function in mediating polarity of cell migration.

## Introduction

**Fig 1:**
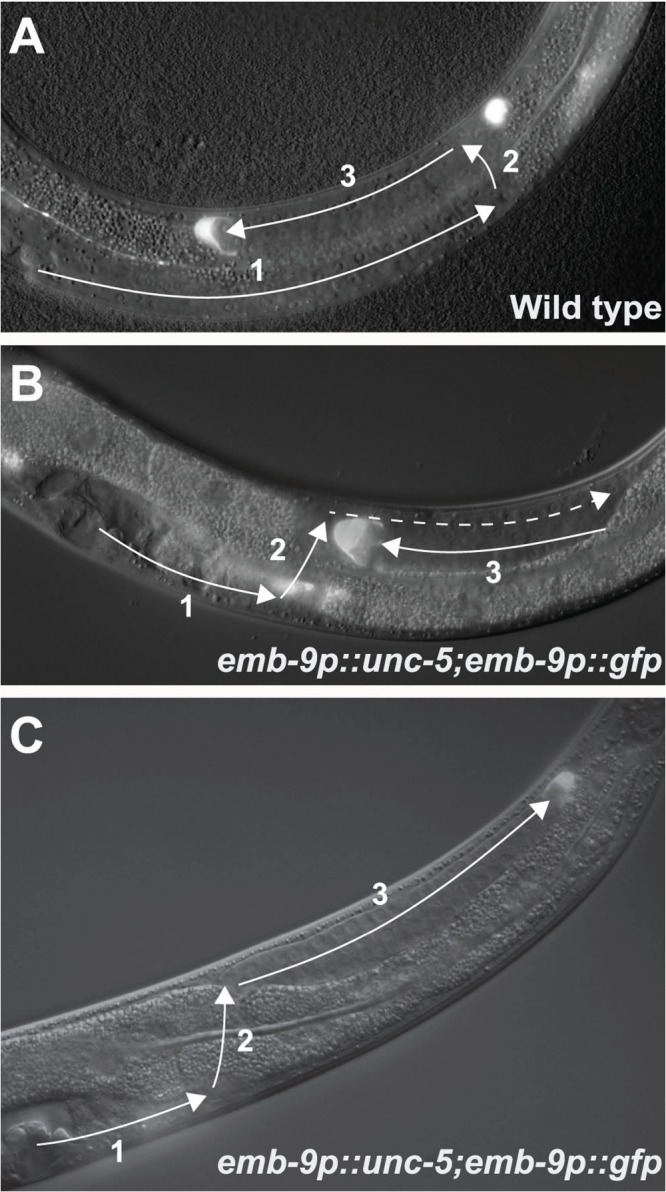
Over-expression of the UNC-6/Netrin receptor UNC-5 causes DTC phase 3 A/P polarity reversals. DIC images of posterior gonad arms in L4 stage hermaphrodites overlaid with florescence images of GFP labeled DTCs. A DTC is located at the tip of each gonad arm. The migratory route taken by the DTC is depicted (white arrows). In all panels anterior is left and dorsal is up. (A) In the wild type, anterior and posterior U-shaped gonad arms are formed by 3 sequential migratory phases (labeled 1, 2, 3 accordingly) of the DTCs. Only the posterior gonad arm and DTC (visualized by the *gly-18p*::*gfp* reporter) are shown. (B) In *evIs129[emb-9p*::*unc-5(+); emb-9p*::*gfp]* animals the anterior or posterior (shown) DTC or both frequently migrates precociously towards the dorsal side. The first migratory phase is then completed on the dorsal muscle band and with normal timing the third migratory phase is initiated, reorienting the DTC back to the mid-body (left edge of photo). Dashed line represents the gonad segment formed in phase 1 that overlaps the segment formed in phase 3. (C) In *evIs129* animals, in addition to the precocious migration of the DTC towards the dorsal side, the anterior or posterior DTC (marked by the *emb-9p*::*gfp* reporter) or both frequently exhibits an A/P polarity reversal that fails to reorient back to the mid-body.

Cell migrations play a central role in both development and pathogenesis. However, the mechanisms underlying guided cell migration, and in particular the means by which extracellular information is integrated within the cell, are poorly understood. The *C*. *elegans* Distal Tip Cells (DTCs) provide an excellent model system to study various regulatory aspects of cell migration. In the hermaphrodite, a DTC is found at the extending tip of each of two elongating hermaphrodite gonad arms. These cells are born post-embryonically in the ventral mid-body and migrate along a stereotyped path involving 3 stages of polarized movements along A/P and D/V axes. In phase 1 the DTCs migrate away from each other along the A/P axis. In phase 2 the DTCs turn 90° and migrate along the D/V axis from the ventral to dorsal body wall muscles. In phase 3, the DTCs again turn 90° and migrate along the A/P axis back to the dorsal mid-body. This migration path determines the U shape of the two symmetrical gonad arms in wild-type animals (Fig 1A). Thus, by monitoring DTC movements in real time or gonad shapes in developed wild type and mutant hermaphrodites, insights may be gained into the regulation of sustained polarized migration over a single axis or transitions from one axis to another, which require the coordinated output of multiple guidance signaling pathways.

Several highly conserved genes have been found to be involved in the regulation of the different aspects of DTC migration [1]. We have discovered that UNC-6/Netrin and Wnt signaling redundantly regulate the guidance of these cells [2]. Functioning together, Netrin and Wnt signaling orchestrate the migratory transitions of the DTCs between the A/P and D/V axes [2]. Here we begin to reveal how migrating DTCs tune their response to these two signaling mechanisms at a molecular level to guide DTC migration.

The UNC-6/Netrin guidance cue and its receptors, UNC-5 and UNC-40/DCC are highly conserved in invertebrates and vertebrates and play central roles in cell and axon guidance. UNC-6 is expressed selectively at the ventral side of the developing animal [3]. This expression pattern predicts a D/V graded distribution of UNC-6 during all phases of DTC migration, which imparts to UNC-6 an ability to provide polarity information needed to guide migrations along the D/V axis of the body wall. Response to this polarity information is mediated by the UNC-5 and UNC-40 receptors.

*unc-5* was found to be transcriptionally activated at the onset of phase 2 just prior to the first 90° turn of the DTC from the A/P to the D/V axis. It was also shown that multi-copy arrays of *unc-5* designed to drive precocious over-expression of UNC-5 in the DTCs caused precocious ventral to dorsal migration of these cells. This precocious migration suggests that increased function of UNC-5 in the DTCs is both necessary and sufficient to drive the transition from the A/P to the D/V axis [4]. Nevertheless, the increase in *unc-5* levels at the initiation of phase 2 is apparently transient as levels of an *unc-5* transcriptional reporter appeared to decrease during phase 3 [4]. Here we show that interfering with the regulation of UNC-5 by over-expression causes A/P polarity reversals during the third migratory phase. This suggests that although an increase in UNC-5 levels at the beginning of phase 2 is required to induce reorientation to the D/V axis, a decrease in *unc-5* function during or prior to transitioning back to the A/P axis is required for proper orientation of DTC phase 3 migration along that axis [2].

Our goal then became to identify the mechanism or mechanisms responsible for this phase 3 regulation of UNC-5. Loss-of-function (lf) mutations in several genes have been reported to induce phase 3 A/P DTC polarity reversals that are phenotypically similar to those induced by overexpressing UNC-5 in the DTCs [5–10]. Among these are genes with mammalian homologs encoding small GTPases (*ced-10/rac* and *mig-2/rhoG*) [6] and small GTPase co-regulators (such as *ced-2/crkII*, *ced-5/dock180* and *ced-12/elmo)*. These small GTPase regulators function together in a signaling pathway that activates CED-10/Rac [11] to regulate apoptotic cell engulfment and DTC migration [7–11]. Cabello et al., subsequently reported that this pathway is activated by MOM-5/Frizzled [5], implicating MOM-5/Frizzled in a non-canonical Wnt signaling pathway mediated by the activation of CED-10/Rac [5,12].

Our previous findings indicate that UNC-6/Netrin, Wnts, and small GTPases have shared functions in both A/P and D/V guidance, and that small GTPases can function as upstream regulators of guidance cue receptors [2,13]. We hypothesized, therefore, that MOM-5 functions through small GTPases to negatively regulate UNC-5 function in phase 3 migration. Here we show that *unc-5* deficits markedly suppress the DTC phase 3 A/P polarity reversals caused by mutations in *mom-5/frizzled*, *ced-12/elmo*, *ced-10/rac*, and *mig-2*/*rhoG*. This suggests that MOM-5/Frizzled normally functions through small GTPases like CED-10 and MIG-2 to negatively regulate *unc-5*. We provide additional evidence to show that *mom-5/frizzled* function is not only necessary, but also sufficient to negatively regulate an *unc-5*-dependent DTC dorsal migration phenotype and to induce D/V axon guidance defects like those of the *unc-5* null. Furthermore, consistent with its suggested role as a negative regulator of *unc-5*, we show that *mom-5/frizzled* expression is up-regulated in the DTCs when *unc-5* down-regulation is apparently required to prevent DTC phase 3 A/P polarity reversals. We conclude that the UNC-5 receptor is a downstream negatively regulated target of the non-canonical Wnt signaling pathway triggered by MOM-5/Wnt activation and mediated by small GTPase signaling involving CED-12/ELMO, CED-10/Rac and MIG-2/RhoG. This reveals a previously unknown regulatory link between components of Wnt and Netrin signaling pathways and illuminates how guidance signaling mechanisms can be integrated to orchestrate directed cell migration.

## Results

### Over-expression of UNC-5 in the DTCs causes phase 3 A/P polarity reversals

**Fig 2:**
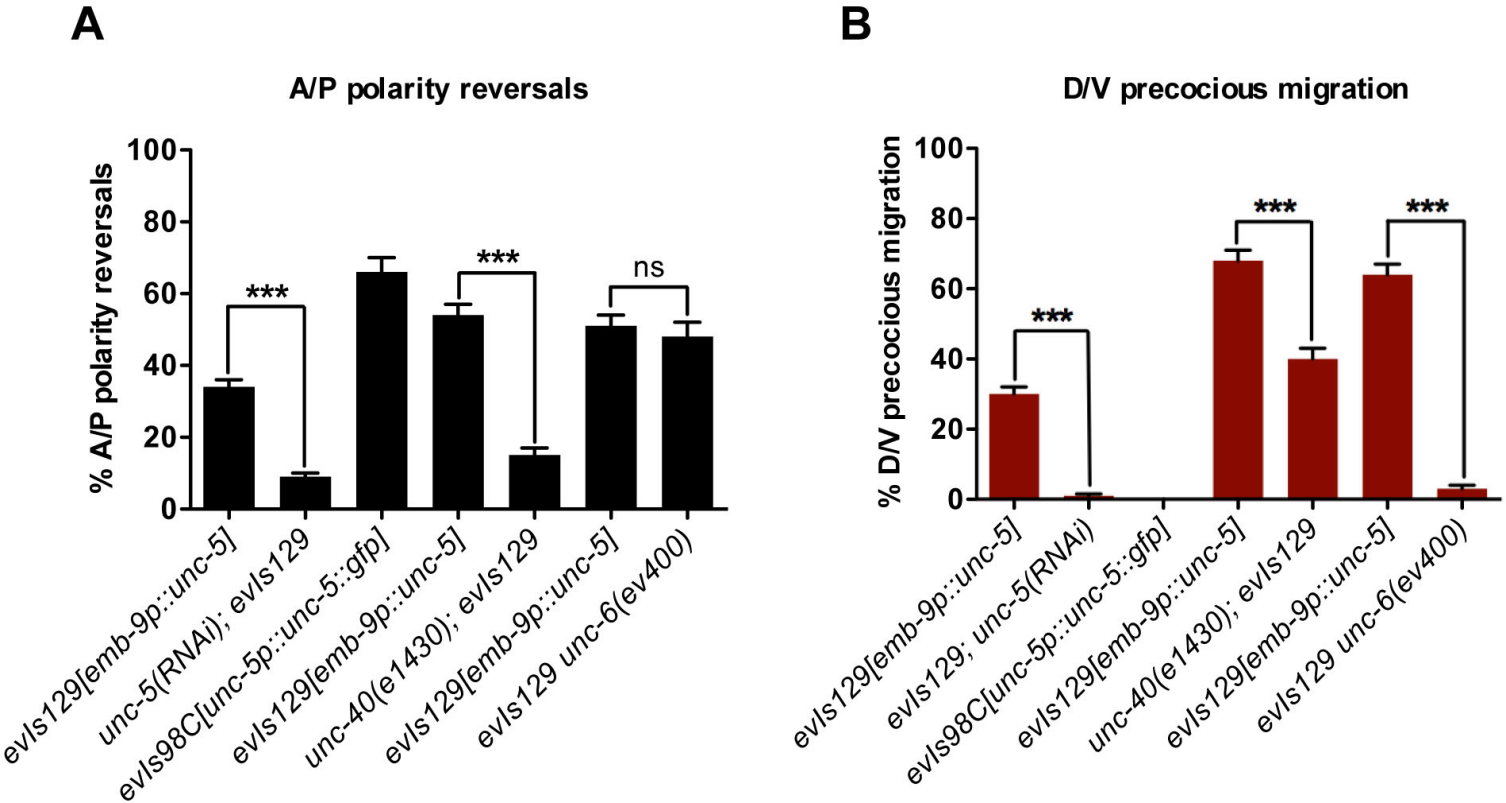
The *evIs129[emb-9p*::*unc-5]* phase 3 A/P polarity reversals are dependent on *unc-5* and *unc-40*, but independent of *unc-6/netrin*. (A) Quantification of phase 3 A/P polarity reversals in *evIs98C* (a multi-copy transgenic array of *unc-5(+)* regulated by its own promoter) or *evIs129* (another multi-copy transgenic array of *unc-5(+)* driven by the *emb-9* promoter) versus *evIs129* treated with *unc-5(RNAi)* or in the background of *unc-40* or *unc-6* null alleles. Bars represent the percentage of posterior DTC phase 3 polarity reversals. The corresponding raw data are presented in S2 Table. Error bars indicate standard error of the sample proportion. Comparisons of the phase 3 A/P reversals were made between each corresponding pair as indicated by the connecting lines. ***P <0.00001; ns = not significant (P≥0.01). (B) Quantification of phase 2 D/V precocious migrations in the strains presented in panel (A). Bars represent the percentage of posterior DTCs that migrate precociously to the dorsal side as determined by the shape of the gonad arms. The corresponding raw data are presented in S2 Table. Error bars indicate standard error of the sample proportion. Comparisons of the D/V precocious migration were made between each corresponding pair as indicated by the connecting lines. ***P <0.00001; ns = not significant (P≥0.01). The corresponding control for RNAi was grown on empty vector RNAi feeding bacteria.

Our previous studies demonstrated the involvement of *unc-5* in A/P polarity [2,13]. We have also demonstrated that A/P polarity requires a fine balance between the functions of UNC-5 and Wnt signaling components [2]. These results led us to examine the consequences of over-expressing *unc-5* in the DTCs. We analyzed DTC migrations in several transgenic lines that overexpress *unc-5*, including a line carrying *evIs129[emb-9p*::*unc-5(+); emb-9p*::*gfp*] (S1 Table), a transgenic multi-copy array that drives precocious over-expression of *unc-5* in the DTCs and also labels the DTCs with GFP. As anticipated from the phenotypes of related transgenic animals [4], many *evIs129* transgenic animals exhibited precocious migration of the DTCs from the ventral to the dorsal body wall muscles (Figs 1B and 2B, S2 Table). In some *evIs129* animals, phase 3 migrations were executed with normal back-to-mid-body polarity (Figs 1B and 2A, S2 Table). However, in many *evIs129* animals, one or both DTCs exhibited an early or mid phase 3 A/P polarity reversal. Instead of migrating to the mid-body, the DTCs of these animals turned and migrated away from mid-body toward the head (anterior DTC) or tail (posterior DTC) (Figs 1C and 2A, S2 Table).

To determine if the DTC phase 3 A/P polarity reversals induced by *evIs129* depend on *unc-5*, we reduced *unc-5* levels in *evIs129* animals using *unc-5(RNAi)*. *unc-5(RNAi)* suppressed the defects observed in *evIs129*; both A/P polarity reversals (Fig 2A and S2 Table) and precocious ventral to dorsal migrations (Fig 2B and S2 Table). This demonstrates that *unc-5* over-expression and, by implication, *unc-5* over-activity, causes both the A/P polarity defects and the precocious D/V migration observed in *evIs129* animals.

We also analyzed DTC migrations when *unc-5(+)* over-expression was driven by its own promoter as in the *evIs98C[unc-5p*::*unc-5(+)*::*gfp]* multi-copy array (S1 Table). This array is not expected to induce precocious dorsal migration of the DTCs because the endogenous promoter is only up-regulated at the phase 1 to 2 transition [4]. However, we predicted that this array could possibly over-express *unc-5* at the phase 2 to 3 transition to an extent that would still induce phase 3 reversals. As anticipated, this array selectively induced phase 3 A/P polarity reversals (Fig 2A and 2B, S2 Table). These data demonstrate that phase 3 reversals occur independently of precocious D/V migration. Furthermore, they suggest that UNC-5 must be tightly regulated at initiation of both the 2nd and 3rd phases of migration to allow for normal transitions of the DTCs from one migration axis to another, and to determine the direction taken along the subsequent axis of migration.

### DTC phase 3 A/P polarity reversals induced by UNC-5 overexpression depends on UNC-40/DCC but not on UNC-6/Netrin

To further illustrate the function of over-expressed *unc-5* in determining A/P polarity, we examined whether *unc-40* or *unc-6* is required for the A/P polarity reversals observed in *evIs129*. We found that *unc-40* deficits, but not *unc-6* deficits, suppressed the DTC phase 3 A/P polarity defects caused by *unc-5* over-expressing arrays (Fig 2A and S2 Table). The DTC results parallel the results we obtained previously from a related analysis performed on axonal A/P polarity reversals induced by overexpressing *unc-40* in the touch receptor neurons [13]. These A/P polarity reversals were also unaffected by *unc-6* mutations but were dependent on *unc-5*. Thus, higher than normal levels of *unc-5* and/or *unc-40* activity may induce DTC and ALM axon A/P polarity reversals independent of UNC-6/Netrin function, but dependent on the respective Netrin receptor, suggesting that these two receptors function together in A/P guidance as they do in D/V guidance [14–16]. This is unlike the precocious D/V migration phenotype of *evIs129*, which was markedly suppressed by *unc-6* mutations but more refractory to the loss of *unc-40* (Fig 2B and S2 Table).

### MOM-5/Frizzled negatively regulates *unc-5* to allow normal polarity of DTC phase 3 migration

**Fig 3:**
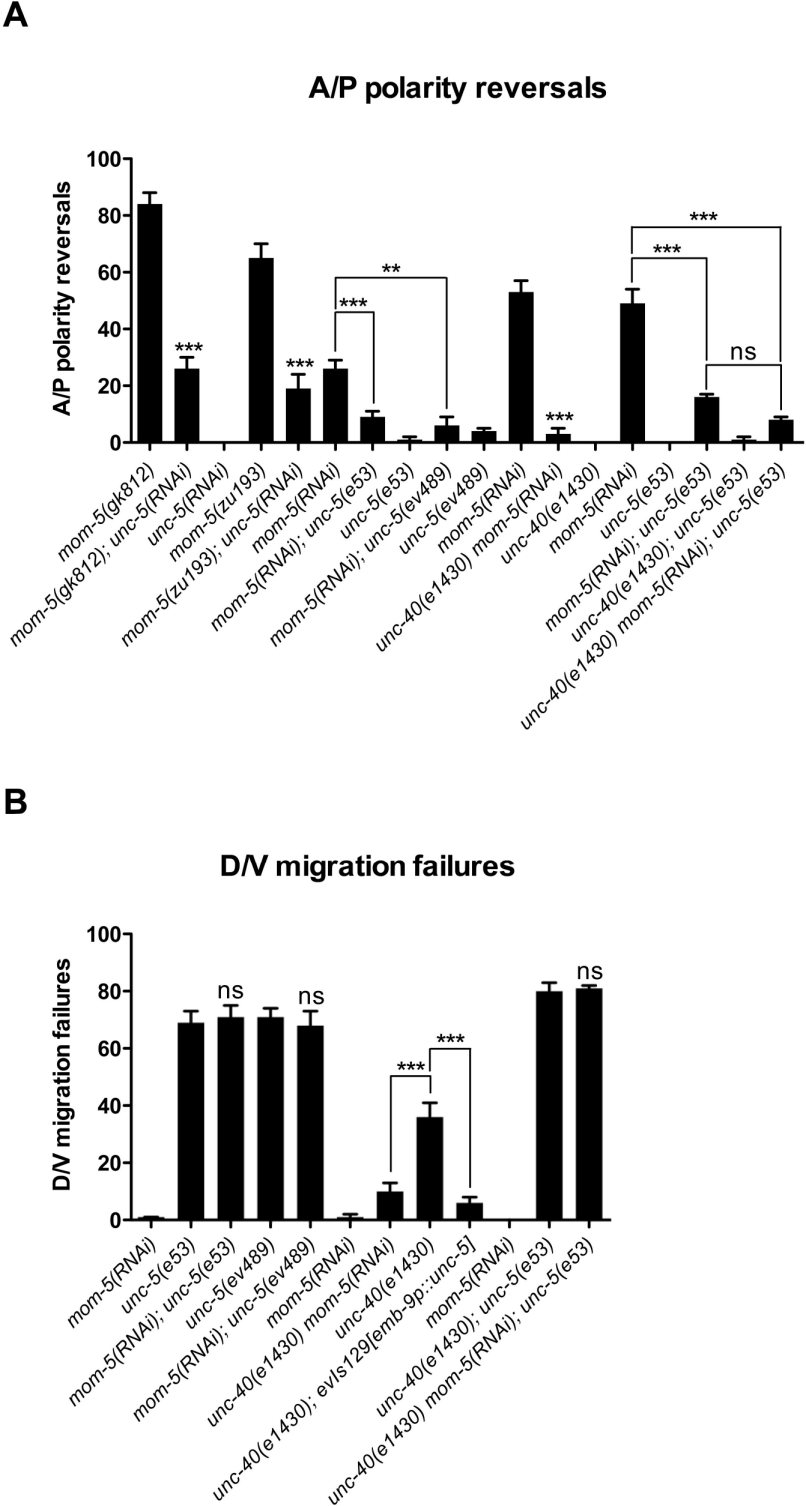
*mom-5* mutant phase 3 A/P polarity reversals are rescued by impairing *unc-5* or *unc-40* function or both, while impairing *mom-5* can rescue *unc-40* D/V defects in a manner that is strictly dependent on *unc-5* function. (A) Percent posterior DTC phase 3 A/P polarity reversals in *mom-5(gk812)* or *mom-5(zu193)* alleles treated or not with *unc-5(RNAi)* plus *unc-5* and *unc-40* single and *unc-40; unc-5* double mutants treated or not with *mom-5(RNAi)*. (B) Percent posterior DTC phase 2 D/V migration failures in *unc-5* and *unc-40* single and *unc-40; unc-5* double mutants treated or not with *mom-5(RNAi)*. Also shown are the effects of *evIs129[emb-9p*::*unc-5]* on DTC phase 2 migration failures of *unc-40(e1430)*. The corresponding raw data including the data for the anterior DTC are presented in S3 Table. Error bars indicate the standard error of the sample proportion. Comparisons of A/P polarity reversals were made between *mom-5* mutant animals treated or not with *unc-5(RNAi)*, or between *mom-5(RNAi)* of N2 compared to *mom-5(RNAi)* of Netrin receptor mutants. Comparisons of D/V defects were made between Netrin receptor mutants treated or not with *mom-5(RNAi)* and *unc-40(e1430)* in the absence or presence of *evIs129*. ***P <0.00001; **P<0.001; ns = not significant (P≥0.01). The corresponding control for RNAi was grown on empty vector RNAi feeding bacteria.

Recently, we showed that UNC-5, like some Wnts and Wnt receptors, functions redundantly with specific Wnts while opposing the function of others to regulate phase 3 A/P polarity of the DTCs [2]. It has also been reported that *mom-5/frizzled* mutants exhibit DTC phase 3 A/P polarity reversals [5]. These polarity reversals are similar to the A/P polarity reversals observed when UNC-5 is over-expressed in the DTCs (Fig 1C). This evidence led us to examine the hypothesis that MOM-5/Frizzled negatively regulates *unc-5* prior to or at the onset of phase 3 migration, thereby allowing normal back-to-mid-body polarity. We tested this hypothesis by reducing *unc-5* function in *mom-5* null mutants to determine if this would rescue the A/P polarity reversals associated with eliminating *mom-5* function. We used *unc-5(RNAi)* to reduce UNC-5’s function in two *mom-5* mutant alleles: a severe lf allele, *mom-5(gk812)*, which is a null for DTC migration (see below), and the putative null allele, *mom-5(zu193)* [5]. In both *mom-5* mutant strains, reducing *unc-5* function caused a marked suppression of the *mom-5* mutant A/P polarity reversals. Reversals decreased in the posterior DTC from a frequency of 65–85% down to 20–25% (Fig 3A and S3 Table). Significant suppression was also observed for the anterior DTC (S3 Table). Similarly, A/P polarity reversals induced by *mom-5(RNAi)* were markedly reduced for the posterior DTC in *unc-5* mutant strains (*e53* or *ev489* alleles) compared to the wild type N2 strain (Fig 3A and S3 Table). This suppression indicates that the A/P polarity reversals caused by depleting *mom-5* function are dependent on *unc-5* function. A likely explanation for the above results is that MOM-5 normally negatively regulates *unc-5* at the onset of phase 3 in order to allow for the back to mid-body reorientation of the DTCs.

It should be pointed out that the use of null alleles is key to this analysis for two main reasons: First, it reduces the likelihood of sheer competition between separate MOM-5 and UNC-5 pathways on a common downstream target (one pathway activating and the other inhibiting the target) as the root cause of the observed suppression. Second, if two genes are found to function in the same pathway with one effectively inhibiting the other, null alleles help determine the hierarchy of gene action. However, *mom-5* mutants are maternally rescued embryonic lethals, so homozygotes must be derived from heterozygous mothers and could therefore harbor maternally inherited wild type *mom-5* function that might still contribute to DTC phase 3 migration. To examine the likelihood of this possibility, we treated wild type and *mom-5* mutants with *mom-5* RNAi to further deplete any possible maternal product and found that *mom-5(RNAi)* induced significant phase 3 A/P polarity defects in the wild type, but failed to increase the penetrance of *mom-5(gk812)* null mutant phase 3 defects (S1A Fig). This suggests that *mom-5(gk812)* is a true null for *mom-5* function in DTC phase 3 migration.

If indeed a *mom-5* deficit causes *unc-5* over-activity one would expect *unc-40* mutations to suppress the DTC A/P polarity reversals caused by a *mom-5* deficit, just as these mutations suppress the A/P polarity reversals caused by overexpressing UNC-5 (Fig 2A and S2 Table). We found that an *unc-40* null mutation suppressed the A/P polarity reversals induced by *mom-5(RNAi)* (Fig 3A and S3 Table). This suppression was as pronounced as when both *unc-5* and *unc-40* were depleted simultaneously compared to either single mutant (Fig 3A and S3 Table), suggesting that *unc-5* and *unc-40* function together in the same pathway to regulate the phase 3 A/P polarity of the DTCs.

### A *mom-5* deficit rescues DTC phase 2 D/V migration failures of an *unc-40* null in an *unc-5* dependent manner

Although *unc-5* mutations markedly suppressed the A/P polarity reversals of *mom-5* mutants, *mom-5(RNAi)* had no effect on the frequency of phase 2 D/V migration failures of *unc-5(e53)* or *unc-5(ev489)* (Fig 3B and S3 Table). However, *mom-5(RNAi)* rescued the posterior DTC phase 2 D/V defects of an *unc-40* null mutant, but not the phase 2 D/V defects of an *unc-40; unc-5* double null (Fig 3B and S3 Table). Thus, the rescue by a *mom-5* deficit of *unc-40* mutant phase 2 D/V guidance is strictly dependent on *unc-5* function. Notably, *unc-5* over-expression in the DTCs also rescued *unc-40* null D/V phase 2 defects (compare *unc-40(e1430)* to *unc-40(e1430); evIs129* in Fig 3B and S3 Table). These results support a model in which a *mom-5* deficit causes an increase in *unc-5* function during phase 2 migration, and that this increase in *unc-5* function rescues the *unc-40* null phase 2 D/V migration failures.

### *ced-12/elmo*, *ced-10/rac*, and *mig-2/rhoG* negatively regulate *unc-5* to allow normal polarity of DTC phase 3 migration

It has also been reported that mutants of *mig-2/rhoG*, *ced-10/rac*, *ced-2/crk-II*, *ced-5/dock180* and *ced-12/elmo* all exhibit DTC phase 3 A/P reversals [5–9,17]. Published work by Cabello et al [5] supports the notion that MOM-5/Frizzled functions upstream of CED-10/Rac to prevent DTC phase 3 polarity reversals [5]. Furthermore, we and others reported the involvement of small GTPases as putative upstream regulators of guidance cue receptors [13,18,19]. Together, these reports raise the possibility that the activation of CED-10/Rac by CED-2,-5, and -12 negatively regulates *unc-5* at the onset of phase 3 to allow for normal back-to-mid-body DTC orientation. In this case reducing *unc-5* activity should suppress the DTC phase 3 A/P polarity reversals observed in mutants of *ced-10/rac*, *mig-2/rhoG* and *ced-12/elmo*.

**Fig 4:**
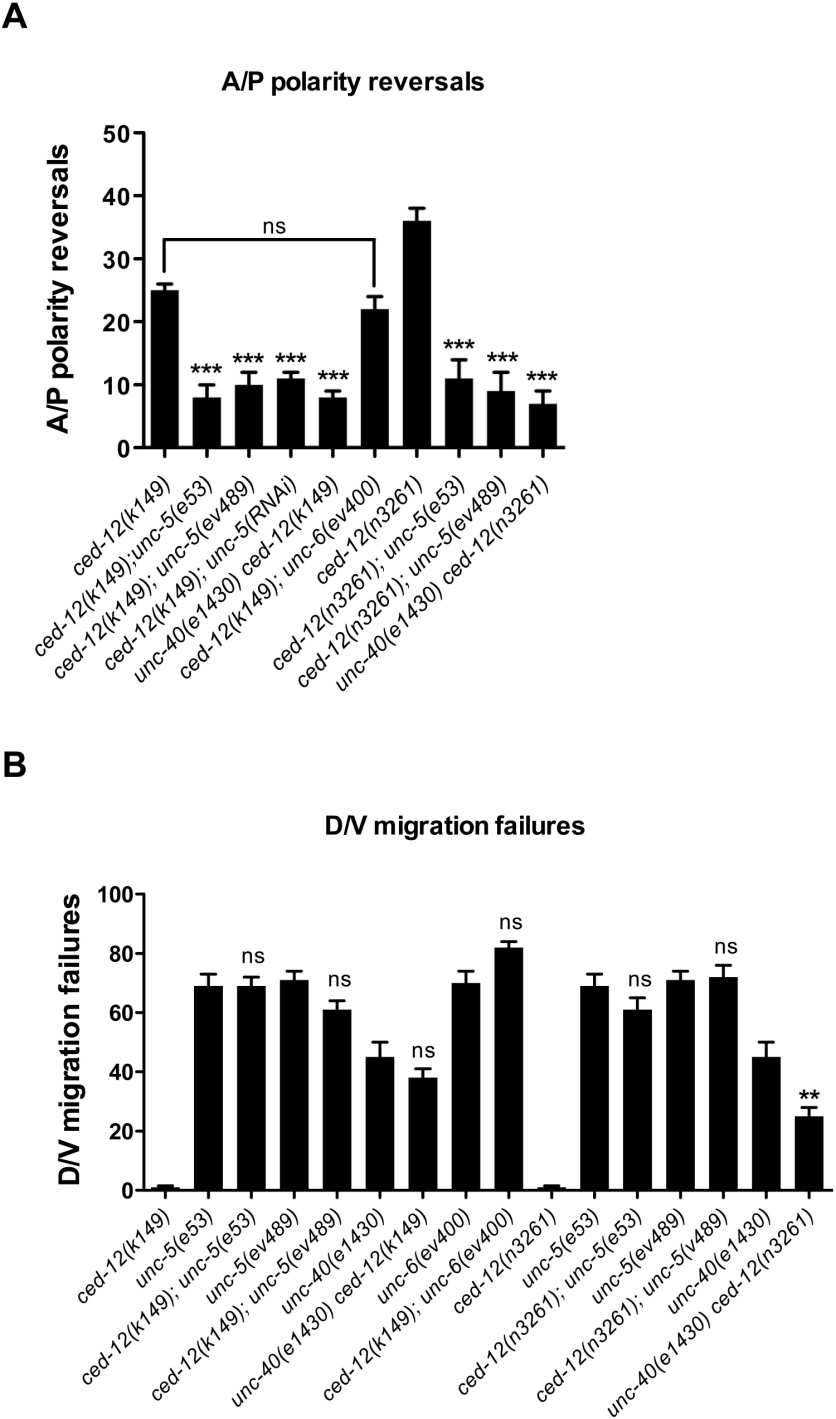
*ced-12* mutant phase 3 A/P polarity reversals are dependent on *unc-5* and *unc-40*, but independent of *unc-6/netrin*. (A) The frequency of posterior DTC phase 3 A/P polarity reversals in single *ced-12(k149)* or *ced-12(n3261)* mutants versus double mutants of *ced-12* with mutants of various UNC-6/Netrin signaling components. (B) The frequency of posterior DTC phase 2 D/V migration failures of *unc-5*, *unc-40*, and *unc-6* mutants and double mutants of these with *ced-12* mutations. The corresponding raw data—including the data for the anterior DTC—are presented in S4 Table. Error bars indicate the standard error of the sample proportion. Comparisons of A/P polarity reversals or D/V migration failures were made between paired single and double deficits. ***P <0.00001 **P<0.001; ns = not significant (P≥0.01).

*ced-12* encodes the *C*. *elegans* homolog of vertebrate ELMO, which functions with CED-2/CrkII and CED-5/DOCK180 as an effective Guanine Nucleotide Exchange Factor (GEF) for CED-10/Rac [7–9,11]. We generated double mutants carrying either of two different alleles of *ced-12* (e.g. *k149* and *n3261*), plus either of two different alleles of *unc-5* (*e*.*g*. *ev489* and *e53*). In all double mutant combinations, an *unc-5* mutation partially but markedly suppressed the posterior A/P polarity reversals caused by a *ced-12* mutation (Fig 4A and S4 Table). Furthermore, *unc-5(RNAi*) reproduced this suppression (Fig 4A and S4 Table). These results are consistent with the possibility that a normal role of CED-12/ELMO, like the proposed role of MOM-5/Frizzled, is to negatively regulate the UNC-5 receptor at the initiation of or during phase 3 to allow normal polarity establishment and back-to-mid-body orientation of the DTCs.

If indeed over-activity of *unc-5* is responsible for the A/P polarity reversals observed in *ced-12* mutants, then these polarity reversals should, like over-expressed *unc-5*, depend on *unc-40* but not on *unc-6*. To test this we generated double mutants of *ced-12* with *unc-40* and with *unc-6* null alleles. The *unc-40* null mutation suppressed the A/P polarity reversals of *ced-12* mutants, whereas the *unc-6* null mutation had no significant effect on these A/P polarity reversals (Fig 4A and S4 Table). These results provide further support to the hypothesis that *unc-5* is effectively overactive in *ced-12* mutants and that the normal function of CED-12/ELMO is to negatively regulate UNC-5 during phase 3 DTC migration. The observation that both *mom-5(RNAi)* and *ced-12(k149)* enhance the A/P polarity reversals of *evIs129* (S2 Fig) is consistent with this model.

Similar to what we observed for *mom-5* mutations, *ced-12* mutations had no effect on the phase 2 D/V migration failures of posterior DTCs in the *unc-5* mutants (Fig 4B and S4 Table) and even enhanced the anterior DTC phase 2 failures of *unc-5(ev489)* and *unc-40(e1430)* (S4 Table, see below). However, similar to what we observed for *mom-5(RNAi)*, *ced-12* mutations partially rescued the phase 2 D/V defects of the posterior DTCs in an *unc-40* null mutant (Fig 4B and S4 Table). This supports a model in which a *ced-12* deficit causes an increase in *unc-5* function that can partially bypass the need for *unc-40*, just as *unc-5* over-expression by multi-copy transgene arrays can partially bypass the need for *unc-40* to mediate phase 2 D/V guidance. This result supports a scenario in which CED-12/ELMO, like MOM-5, functions as a negative regulator of UNC-5 expression, function, or localization.

**Fig 5:**
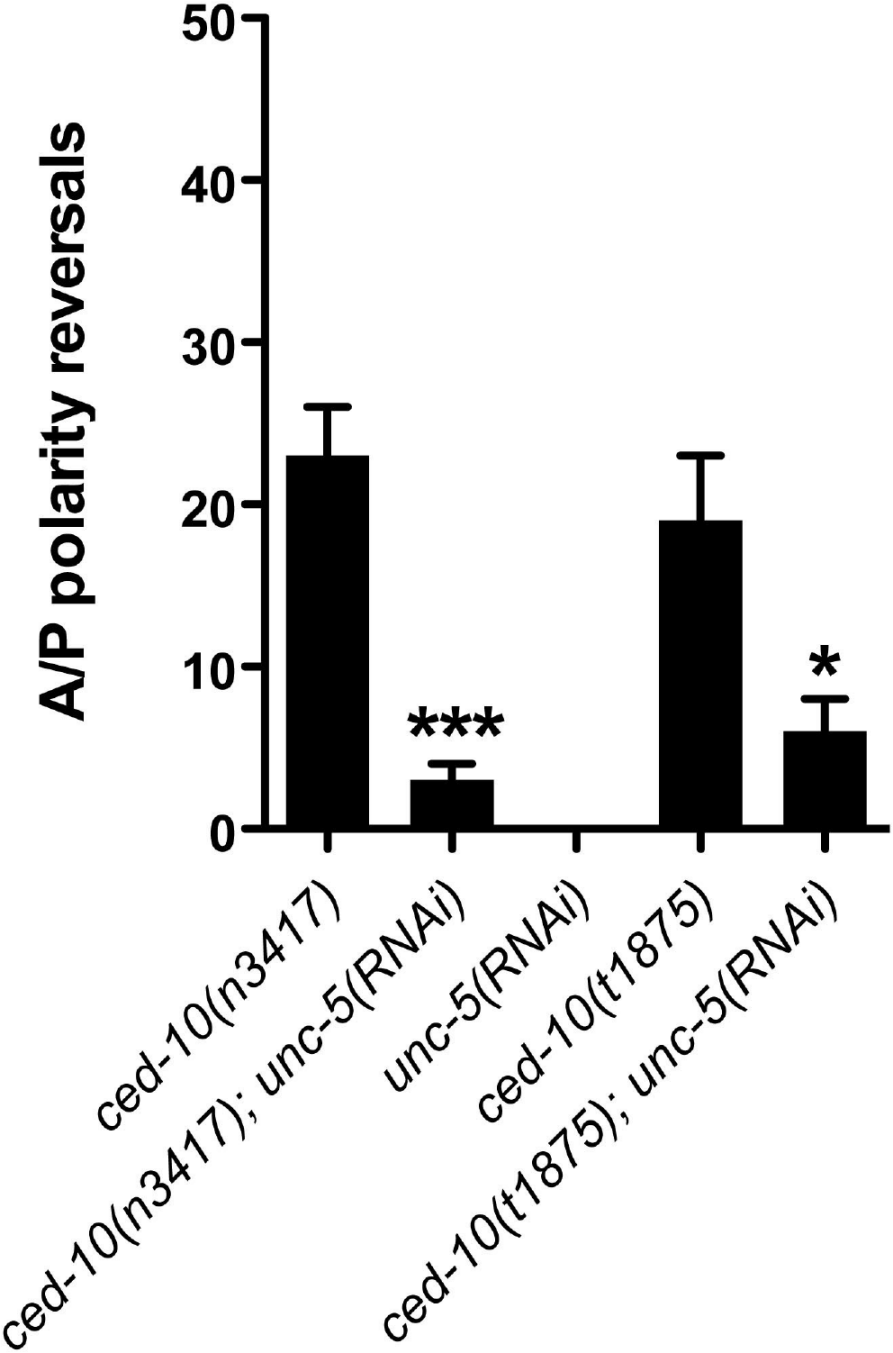
*ced-10* mutant phase 3 A/P polarity reversals are dependent on *unc-5*. The frequency of posterior DTC phase 3 A/P polarity reversals in *ced-10(t1875)* or *ced-10(n3417)* alleles treated or not with *unc-5(RNAi)*. The corresponding raw data including the data for the anterior DTC are presented in S5 Table. Error bars indicate the standard error of the sample proportion. Comparisons of A/P polarity reversals were made between control (empty vector) and *unc-5(RNAi)* treated animals. ***P <0.00001 *P<0.01; ns = not significant (P≥0.01).

CED-10/Rac is activated by a complex of CED-12/ELMO with CED-2/CrkII and CED-5/DOCK180 [7–9,11] and is expressed in the DTCs [6]. *unc-5(RNAi)* significantly suppressed A/P polarity reversals of the posterior DTC in a *ced-10(n3417)* severe lf mutant and the putative null mutant, *ced-10(t1875)* (Fig 5 and S5 Table). This suggests that *ced-10/rac* also functions as a negative regulator of *unc-5* to determine normal polarity of DTC phase 3 migration.

**Fig 6:**
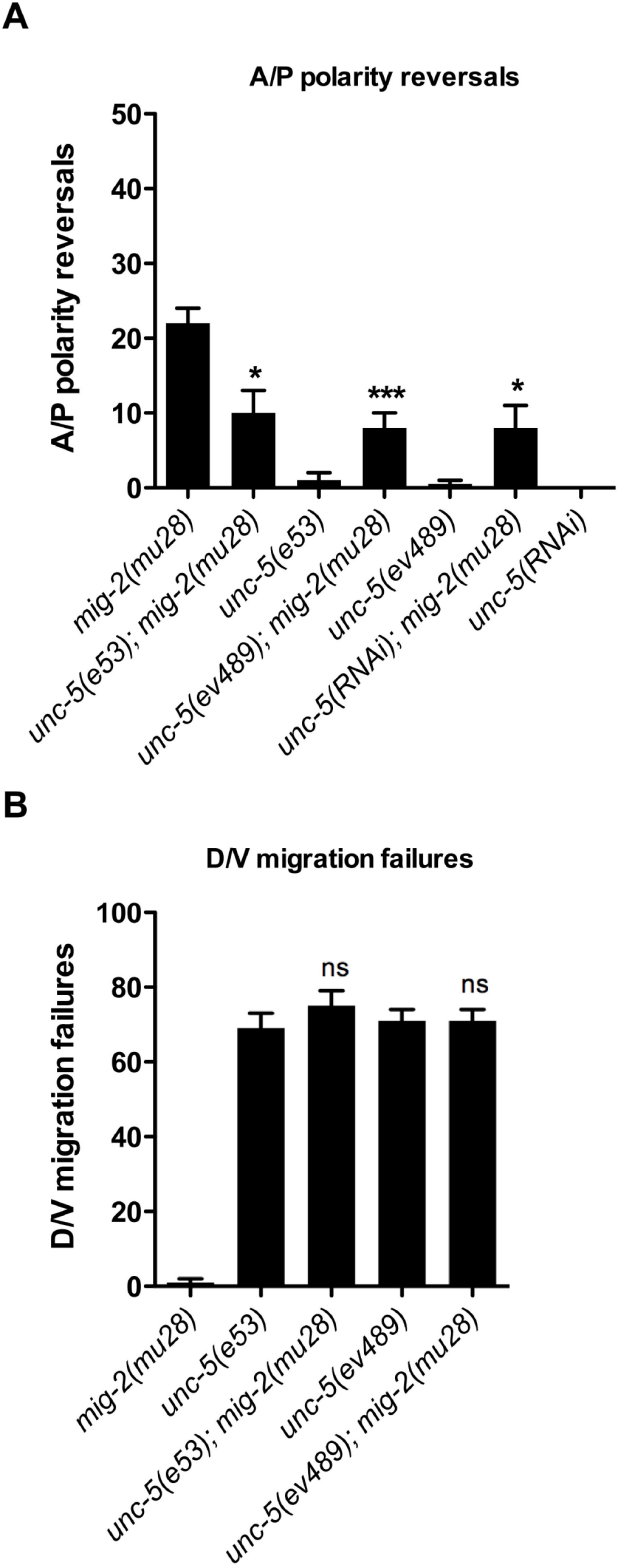
*unc-5* mutations suppress *mig-2(mu28)* mutant DTC phase 3 A/P polarity reversals, but *mig-2(mu28)* does not suppress *unc-5* mutant phase 2 D/V migration failures. (A) The frequency of posterior DTC phase 3 A/P polarity reversals in single *mig-2(mu28)* mutants versus double mutants of *mig-2(mu28)* with various *unc-5* null alleles or *unc-5(RNAi)*. (B) The frequency of posterior DTC phase 2 D/V migration failures in double mutants of *mig-2(mu28)* with *unc-5 (e53* and *ev489* alleles) versus the respective *unc-5* single mutants. The corresponding raw data, including the data for the anterior DTC, are presented in S6 Table. Error bars indicate the standard error of the sample proportion. Comparisons of A/P polarity reversals or D/V migration failures were made between paired single and double deficits. ***P <0.00001 *P<0.01; ns = not significant (P≥0.01).

We also tested the effects of *unc-5* loss of function on the A/P polarity reversals of *mig-2/rhoG* mutants. *mig-2/rhoG* functions partially redundantly with *ced-10/rac* in other contexts [6,20,21], was implicated as an upstream regulator of CED-12/ELMO in apoptotic cell engulfment [11] and is expressed in the DTCs [22] throughout their migration (S3 Fig). Two alleles of *unc-5* (*e53* and *ev489*) and *unc-5(RNAi)* significantly suppressed the posterior DTC A/P polarity reversals of *mig-2(mu28)* null allele (Fig 6A and S6 Table). Similar to what we observed with *ced-12* mutant alleles and *mom-5(RNAi)*, the penetrance of D/V phase 2 migration failures in *unc-5; mig-2* double mutants were not significantly different from those of *unc-5* mutants (Fig 6B). However, *mig-2(mu28)* selectively enhanced the defects of the *unc-5(ev489)* allele in the anterior DTC (S6 Table). This recurring anterior enhancement is not entirely surprising given that the A/P polarity of anterior and posterior DTC phase 3 migrations have different requirements for Wnt signaling. Some Wnts function redundantly with UNC-5 in the context of anterior but not posterior DTC migration and thus these Wnts could utilize MIG-2, CED-12, or both to regulate phase 3 A/P polarity in anterior DTC migration [2].

### A *mig-2* gain-of-function mutation induces DTC D/V phase 2 failures that are rescued by a transgene array expressing *unc-5(+)* in the DTCs

**Fig 7:**
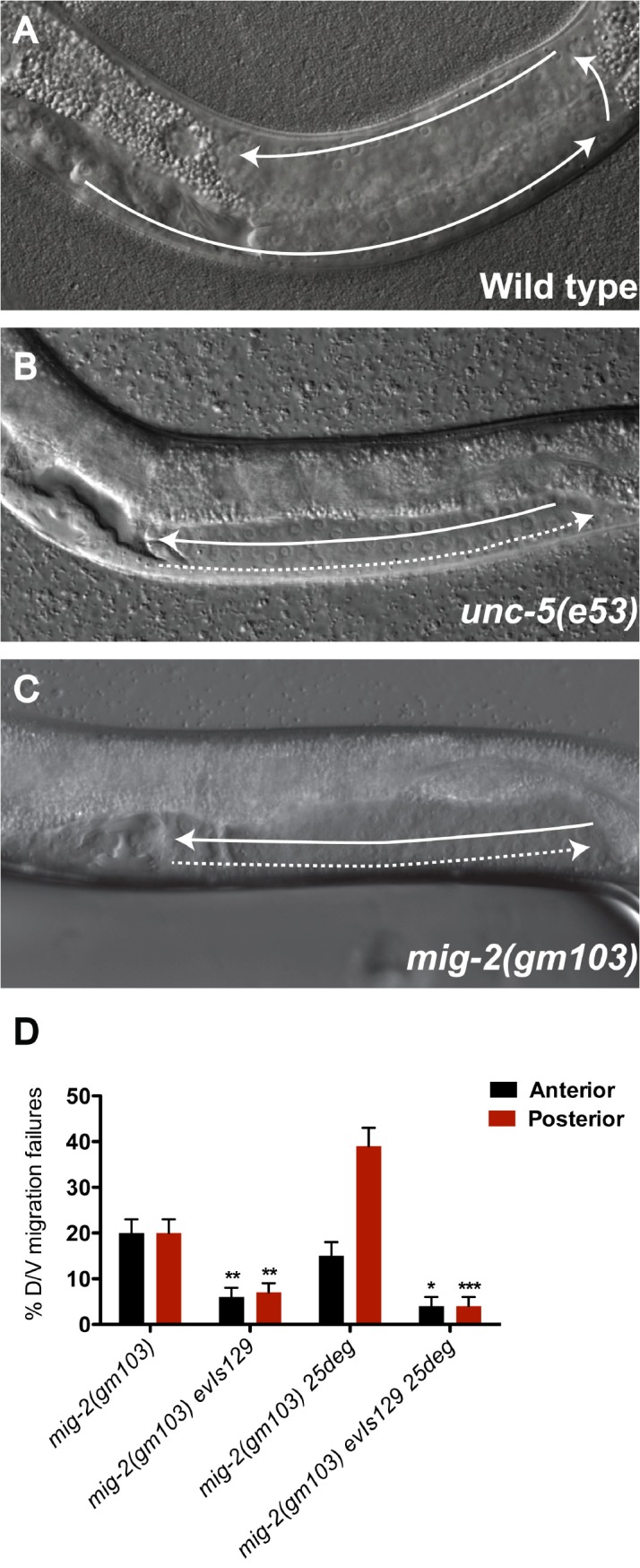
The constitutively active *mig-2(gm103)* gain-of-function allele displays *unc-5* mutant-like phase 2 D/V migration failures suppressed by over-expressing *unc-5(+)* in the DTCs. (A-C) DIC images of posterior gonad arms in L4 stage hermaphrodites. In *mig-2(gm103)* the anterior or posterior (shown) DTC or both (C), like *unc-5(e53)* (B), fails to execute the phase 2 D/V migratory phase and generates ventralized gonad arms. (D) Phase 2 failures of *mig-2(gm103)* are significantly suppressed by *evIs129*, which over-expresses *unc-5* in the DTCs. The corresponding raw data, including the data for the anterior DTC, are presented in S6 Table. Error bars indicate the standard error of the sample proportion. Comparisons of phase2 D/V migration failures were made between paired *mig-2(gm103)* and *mig-2(gm103) evIs129* doubles. ***P <0.00001; **P<0.001; *P<0.01.

In further support for the proposed role of small GTPases as negative regulators of the UNC-5 receptor, *mig-2(gm103)*, a constitutively active gain-of-function (gf) allele of *mig-2/rhoG* [22], causes defects typical of *unc-5* mutants. *mig-2(gm103)* worms are severely uncoordinated and display DTC migration defects, which include DTC phase 2 D/V migration defects (Fig 7C and S6 Table). The *unc-5* transgene *evIs129* can rescue the DTC migration defects of *mig-2(gm103)* animals (Fig 7D). This rescue demonstrates that the DTC defects of this *mig-2(gf)* allele arise from a reduction or loss of *unc-5* function (S6 Table). The suppression of the DTC phenotype of the *mig-2(gm103)* allele by *unc-5(+)* overexpression leads to the same conclusion obtained by the suppression of a *mig-2(lf)* by an *unc-5* deficit, namely, MIG-2/RhoG (like MOM-5/Frizzled, CED-12/ELMO, and CED-10/Rac) negatively regulates *unc-5* to allow normal polarity of DTC phase 3 migration.

### Ectopic expression of *mom-5(+)* is sufficient to negatively regulate *unc-5* in the DTCs and in neurons

The data shown in Fig 3 suggest that MOM-5 is necessary to negatively regulate *unc-5* in the DTCs during their phase 3 migration. To test whether over-expressing *mom-5* is sufficient to negatively regulate *unc-5* in the DTCs, we used the *gly-18* promoter to drive expression of a *mom-5* transgene in these cells [23]. The *gly-18* promoter drives expression of a GFP reporter in the DTCs beginning prior to their first phase migration and continuing to the adult stage (S4A Fig and Fig 1A). Since *mom-5* expression in DTCs normally appears near the beginning of phase 2 migration (see below), the *gly-18p*::*mom-5* transgene array is likely to cause precocious *mom-5* expression during phase 1. A measure of *unc-5* function during DTC phase 1 migration is provided by precocious over-expression of *unc-5* in these cells (as induced by the *evIs129* array). This precocious over-expression causes precocious ventral to dorsal DTC migration (Fig 2 and S2 Table) that is highly sensitive to the levels of *unc-5* [4]. By creating a line carrying both arrays, we found that *gly-18p*::*mom-5* expression suppressed the *evIs129*-induced *unc-5*-dependent precocious DTC migrations (S5 Fig). This suppression indicates that *mom-5* expression in the DTCs during phase 1 migration is sufficient to negatively regulate *unc-5* in these cells.

As a control, we found that *mom-5* expressed from the *gly-18p*::*mom-5* array is functional since it can largely rescue the phase 3 migration of *mom-5* mutant DTCs (S1B Fig). While *gly-18p*::*mom-5* was sufficient to rescue *mom-5* mutant phase 3 polarity defects and suppress *unc-5* induced precocious migration during phase 1, it was not sufficient to induce phase 2 failures. Apparently the *gly-18p*::*mom-5* array establishes an *unc-5* activity level in wild-type animals that is above the threshold for phase 2 initiation, but below the threshold for precocious *unc-5* dependent ventral to dorsal migration. The apparent difference between phase 1 and phase 2 thresholds is predicted by our previous finding that *unc-5* dependent ventral to dorsal migrations are greatly facilitated at the beginning of phase 2 compared to phase 1 [4], thus the above results are not unexpected.

**Fig 8:**
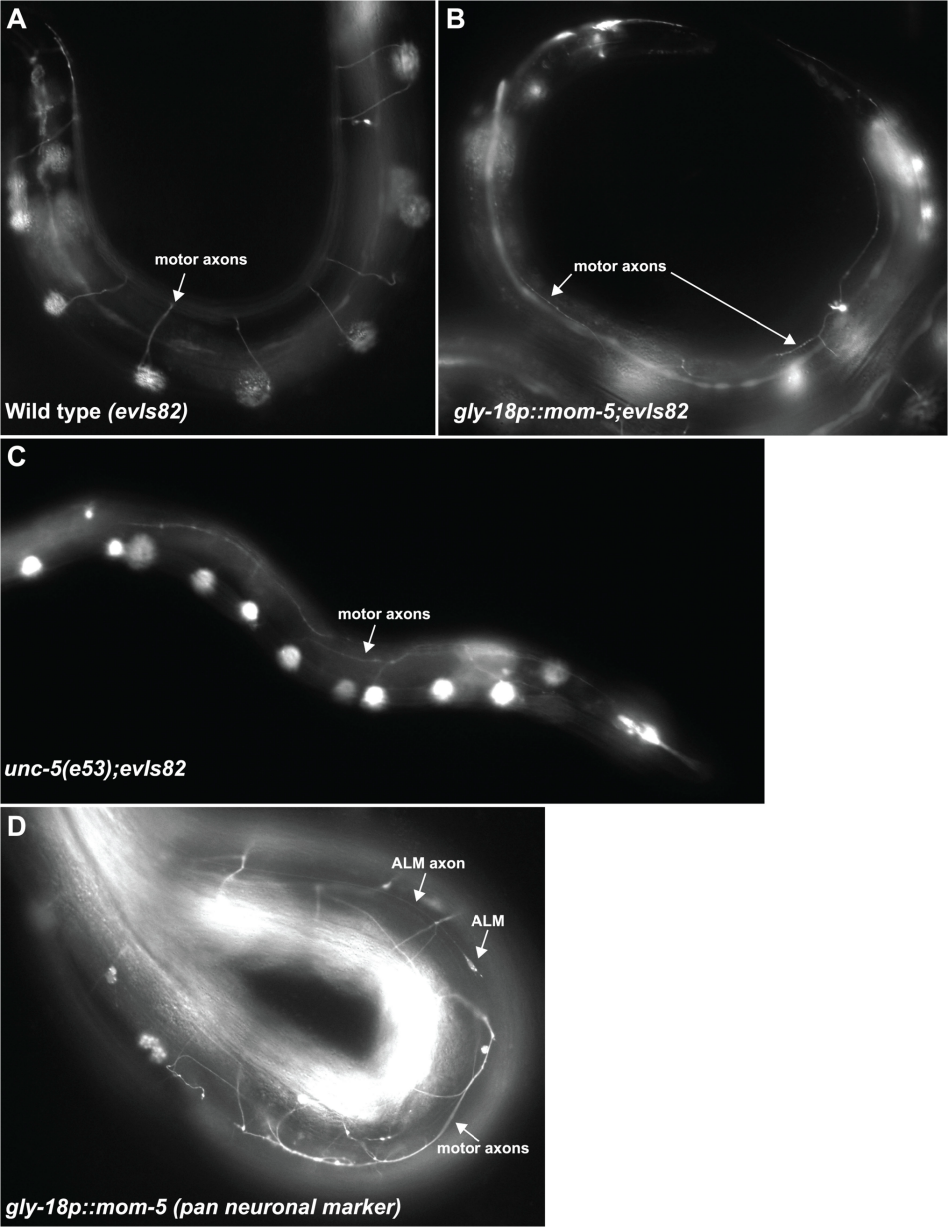
Expression of *mom-5* driven by the *gly-18* promoter induces *unc-5* null-like D/V axon guidance defects of the motor axons. Fluorescence micrographs of hermaphrodites carrying a *gly-18p*::*mom-5* transgenic array. Anterior is left and dorsal is up. (A) In *evIs82[unc-129p*::*gfp]* gfp is expressed in the DA and DB motor neurons, which like other sets of motor neurons, normally extend their axons from the ventral to the dorsal side. The *unc-5* receptor mediates the repulsion of these axons away from the ventral cord in response to the UNC-6/Netrin secreted from ventral neuroblasts. (B) In *gly-18p*::*mom-5; evIs82* the DA and DB motor neuron axons fail to extend to the dorsal side but instead extend in an abnormal lateral position, similar to *unc-5(e53)* null mutant animals (C). (D) In *gly-18p*::*mom-5* animals carrying the pan neuronal reporter *rab-3p*::*gfp* motor axons are misguided while ALM mechanosensory neuron guidance along the A/P axis is normal.

A prevalent phenotype observed in over 30 transgenic lines carrying the *gly-18p*::*mom-5* array was a locomotion defect. All of these transgenic lines segregated paralyzed Uncs resembling the *unc-5* null uncoordinated phenotype. These Uncs exhibited motor axon D/V guidance defects like those of the *unc-5* null (Fig 8). These defects were specific to the motor neurons as guidance of axons such as the ALM axons along the A/P axis was normal in these transgenic lines (Fig 8D). This suggests that while an attempt to over-express *mom-5* in the DTCs was not sufficient to induce phase 2 migration failures, ectopic expression of *mom-5* in other tissues was sufficient to cause D/V axon migration defects and an uncoordinated phenotype similar to that of the *unc-5* null. We detect low expression of a *gly-18* transcriptional reporter in the ventral nerve cord as presented in Supplemental S4B Fig, which could account for this phenotype. It should be noted that *mom-5* expression is not normally detected in the motor neurons, and *mom-5* null mutants are not Unc. The expression of *gly-18p*::*mom-5* in the nervous system is therefore likely ectopic.

### *mom-5* expression in DTCs is up-regulated prior to the phase 2 to phase 3 transition

**Fig 9:**
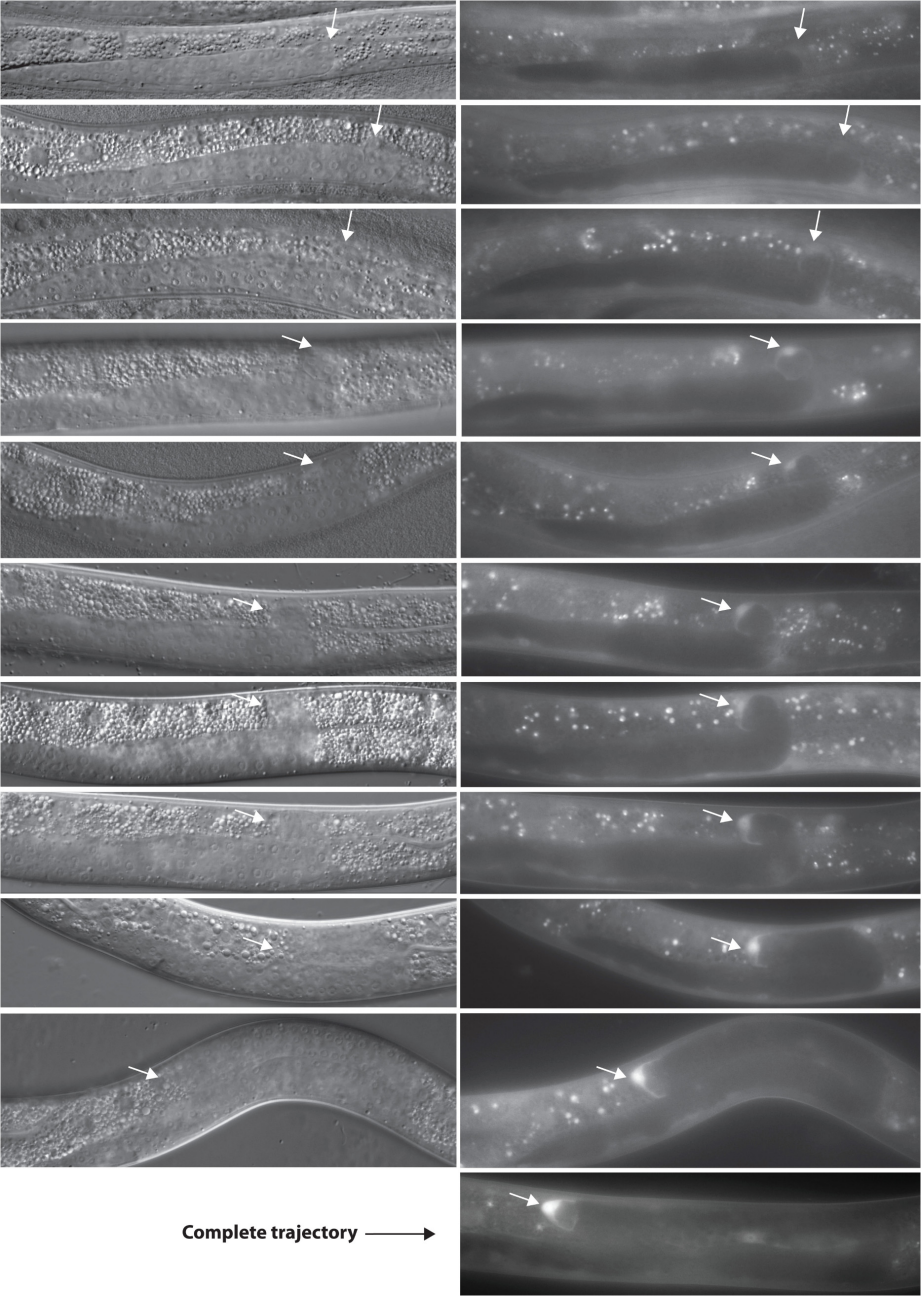
*mom-5* is transcriptionally activated prior to the onset of phase 3. DIC and fluorescence micrographs of hermaphrodites carrying an integrated *mom-5p*::*gfp* single copy transcriptional reporter (*evIs462*). Anterior is left and dorsal is up. Arrows mark the DTC. Sequential stages of DTC migration are shown starting at the onset of phase 2 to the end of phase 3. A single gonad arm is shown. Similar patterns of expression were observed both at the anterior and the posterior DTCs (shown). Gain and exposure time were kept constant for all images presented here. Additional images taken by confocal microscopy were quantified for different phases of DTC migration and the data are presented in S6 Fig.

Our results suggest that *unc-5* function needs to be down-regulated by MOM-5 by the time of the second DTC turn to allow proper DTC phase 3 polarity. In principle, this could be caused, among other things, by a rise in *mom-5* function at or prior to the time of that turn. We therefore examined the expression of a *mom-5* transcriptional GFP reporter generated as a single copy MosSCI insertion. The reporter is undetectable throughout phase 1 (S6 Fig) and begins to be faintly visible in the DTCs during phase 2 immediately after the first turn (Fig 9, top panel) then continues to increase in intensity as the turn occurs and into phase 3 (Fig 9), resulting in an overall 10-fold increase (S7 Fig and S7 Table). Thus, the expression of MOM-5 visibly increases when UNC-5 activity appears to be negatively regulated. These results are entirely consistent with the model favored by all of the above genetic data, which suggests that MOM-5 targets *unc-5* for inhibition in the DTCs at or prior to the beginning of phase 3 migration and this inhibition is required for normal DTC A/P polarity during phase 3.

## Discussion

Exploring the molecular mechanisms that regulate transitions in cell migration from one migratory axis to another can help elucidate how integration of multiple signaling pathways orchestrate polarity of migrating cells, and thus could have broad implications for many aspects of development and tumor progression. In order to identify and characterize these mechanisms, we study the guided migrations of the *C*. *elegans* DTCs, which occur in 3 sequential linear phases along the A/P axis or the D/V axis. We originally discovered a role for UNC-6/Netrin signaling through UNC-5 and UNC-40/DCC receptors in the execution of the DTC phase 2 D/V migration [4,15]. Several reports have suggested that Wnt signaling regulates the A/P polarity of DTC phase 3 migration [5,24,25]. The effect of Netrin signaling on D/V migration and of Wnt signaling on A/P migrations is consistent with the D/V and A/P graded distributions of these guidance cues as these distributions can provide instructive polarity information along these respective axes. In contrast to this graded guidance cue determination of polarity, we recently found that UNC-6/Netrin and Wnt signaling have roles in guiding DTC migrations along axes orthogonal to the axes of their gradation [2]. These findings raise the possibility of cross-talk between Netrin and Wnt signaling mechanisms, which is the subject of the studies reported here. A study of *unc-5* transcriptional reporters showed that these reporters are undetectable during DTC phase 1 migration and become highly visible precisely at the time of the first turn [4]. Furthermore, transgenic multicopy arrays of *unc-5* designed to cause precocious *unc-5* over-expression in the DTCs during phase 1, were found to induce precocious ventral to dorsal DTC migration. This demonstrates that the apparent up-regulation of *unc-5* expression at the time of the first turn is largely causal for that turn [4]. Here we show that transgenic multi-copy arrays of *unc-5* also induce DTC phase 3 polarity defects that are suppressed by *unc-5(RNAi)*. This suggests that some form of down-regulation of *unc-5* activity in phase 3 is necessary for proper A/P polarity of the DTCs. Together, these results suggest that UNC-5 activity levels must be tightly regulated not only for the transition from phase 1 A/P to the phase 2 D/V axis, but also for proper back-to-midbody polarity of phase 3 migrations along the A/P axis.

The effect of multi-copy arrays of *unc-5* on DTC phase 3 A/P polarity is reminiscent of the effect of *unc-40* multi-copy arrays designed to over-express *unc-40* in the touch receptor neurons. We found that the latter arrays caused *unc-5*-dependent A/P polarity reversals of touch receptor axons [13]. Thus, in two different cell types, over-expression of an UNC-6/Netrin receptor causes polarity reversals for migrations (of cells or axons) that occur along the A/P axis. This raises the possibility that normal polarized migration requires signaling pathways that modulate the activity of these receptors at appropriate times during the migration process.

To identify a signaling pathway that regulates *unc-5* during DTC phase 3 migration, we considered the function of the small GTPases MIG-2/RhoG, CED-10/Rac and the co-activator CED-12/ELMO, which usually functions in a complex with CED-2/CRKII and CED-5/DOCK180 [11]. This small GTPase signaling pathway is conserved in vertebrates [11,26–28] and has been shown to be required for apoptotic cell engulfment and guided DTC migrations in *C*. *elegans* [6–8,10,11,17]. Cabello et al reported that this small GTPase signaling pathway is activated by a signal from the MOM-5/Frizzled Wnt receptor [5].

When components of this non-canonical Wnt pathway (MOM-5 –CED-2,-5,-12 –CED-10) are functionally deficient, DTC phase 3 A/P polarity reversals are induced [5]. Here we report that *unc-5* multi-copy arrays overexpressing UNC-5 in the DTCs [4] induce phase 3 A/P reversals that largely mimic the DTC migration defects of *mom-5*, *ced-12*, *ced-10* and *mig-2* mutants. These results raise the possibility that this pathway is involved in the negative regulation of *unc-5* during phase 3 migration, which, as shown here, is necessary for normal DTC phase 3 A/P polarity. We examined this possibility by determining whether *mom-5*, *ced-12*, *ced-10* and *mig-2* mutant DTC migration defects depend on *unc-5* function. We found that impairing *unc-5*’s function significantly suppressed phase 3 A/P polarity reversals found in mutants of *mom-5* and of all the small GTPase signaling molecules tested here (*ced-12/elmo*, *ced-10/rac* and *mig-2/rhoG*). Furthermore, the A/P polarity reversals induced in *ced-12* mutants displayed the same molecular requirements as the A/P polarity reversals induced by over-expressing *unc-5*, as they were dependent on *unc-40/DCC*, but were independent of *unc-6*/netrin function.

Eliminating *unc-40* function could also suppress the phase 3 A/P polarity reversals caused by *mom-5* deficits. This suppression was incomplete and similar in extent to the suppression caused by eliminating *unc-5* function or both *unc-5* and *unc-40* function. These results suggest that although *unc-5* and *unc-40* may function redundantly to some extent to regulate phase 2 D/V migrations, they function largely interdependently to regulate phase 3 A/P migrations. Furthermore, although *unc-5* and *unc-40* are major targets for MOM-5/Frizzled and small GTPase regulation, other MOM-5/Frizzled targets likely exist.

The ability of *unc-5* deficits to suppress phase 3 A/P polarity reversals caused by mutations of the non-canonical Wnt pathway involving *mom-5/frizzled*, *ced-10*, and *ced-12* is consistent, in theory, with either of two different interpretations. One, that the MOM-5-CED-10 pathway normally negatively regulates *unc-5* function, the other, that the MOM-5-CED-10 pathway functions in parallel to UNC-5, with each receptor mediating an opposite outcome (as implicated from their phenotypes i.e. a *mom-5* deficit phenocopies *unc-5* over-expression/activation) and the balance between their outputs is required for normal DTC polarity. Thus when one of the two pathways is compromised, a defect arises, but when both pathways are compromised, the required balance is restored. By this model, a deficit in *unc-5* function could suppress a deficit in *mom-5* function simply by restoring the balance between the output of these two pathways.

**Fig 10:**
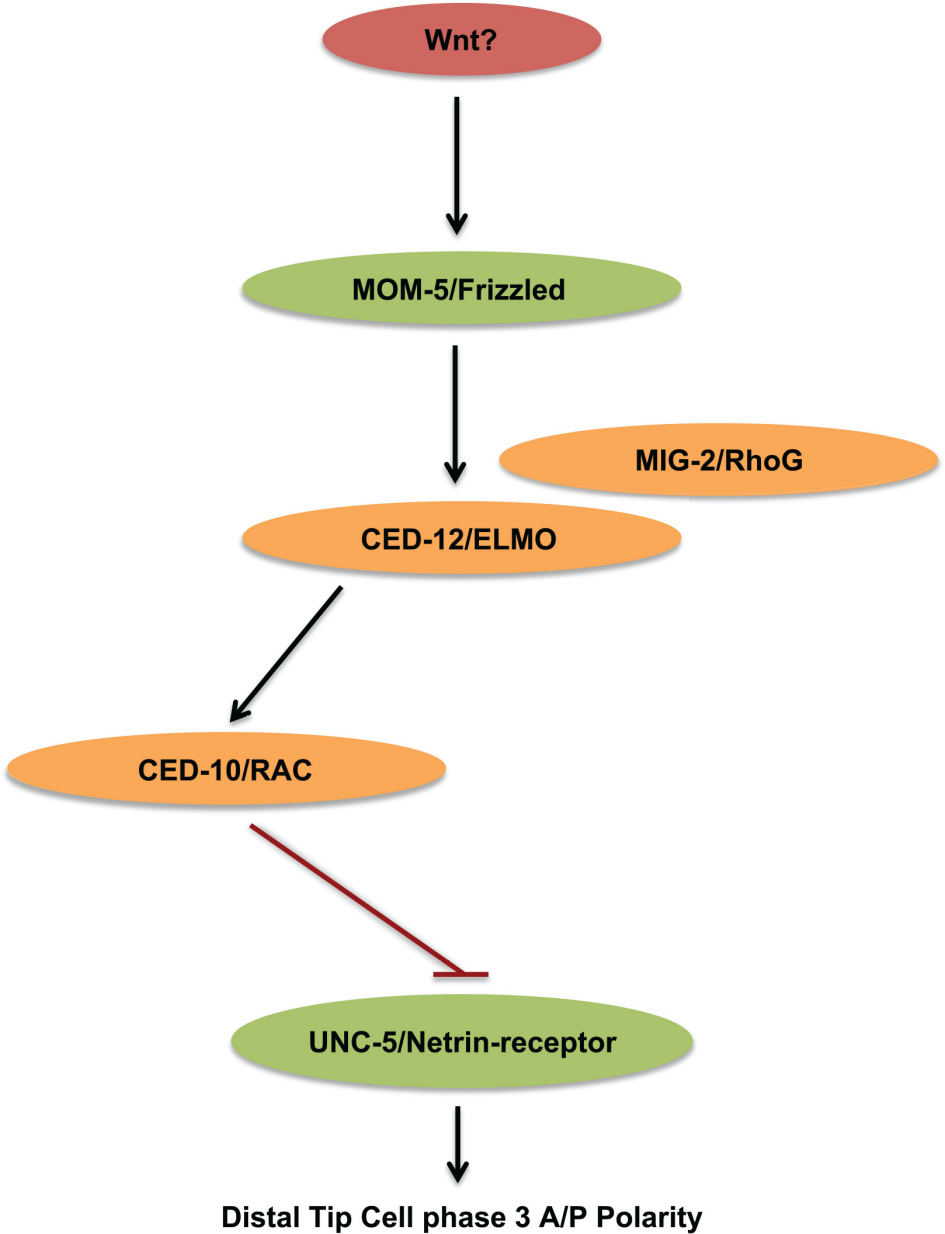
MOM-5/Frizzled through small GTPases functions to negatively regulate the UNC-5 receptor for UNC-6/Netrin. Here, we provide a target for the non-canonical Wnt signaling pathway mediated by the MOM-5/Frizzled receptor, CED-12/ELMO, and CED-10/Rac [5], placing MOM-5 and small GTPases upstream of UNC-5 to negatively regulate *unc-5* and determine the polarity of phase 3 DTC migration. MIG-2/RhoG had been shown to function as an activator of CED-12/ELMO by recruiting the ELMO/DOCK180 complex to the plasma membrane during engulfment of apoptotic cells [11]. It is possible that also in the DTCs MIG-2/RhoG functions upstream of CED-12/ELMO to regulate CED-10/Rac. Ligand in red, small GTPase signaling molecules in orange, and receptors in Green.

These models make different predictions about the effects of alterations in one pathway on the outcome of the other. If *mom-5* and *unc-5* act in parallel pathways and the balance between their outputs is required for normal DTC polarity, then one would expect the suppression to be reciprocal that is, a *mom-5* deficit is expected to suppress the DTC phase 2 failures of an *unc-5* null mutant just as an *unc-5* deficit suppresses the phase 3 A/P polarity defects of a *mom-5* null mutant. However, if *mom-5* functions to negatively regulate *unc-5* by functioning in the same pathway, we would expect a *mom-5* deficit to cause an increase in *unc-5* activity. One read out we have for *unc-5* activity is its ability to induce and mediate DTC phase 2 ventral to dorsal migration. We found that a transgenic array designed to induce *unc-5* over-expression in the DTCs (*evIs129*) could partially rescue the phase 2 defects of an *unc-40* null mutant. This rescue suggests that increased *unc-5* activity can largely compensate for the loss of *unc-40* activity in phase 2 migration. We also found that *mom-5* and *ced-12* deficits rescued the DTC phase 2 D/V migration defects of an *unc-40* null mutant while, in stark contrast, *mom-5* deficits had no effect on the D/V defects in the *unc5* null mutant or the *unc-40; unc-5* double null mutant. This demonstrates that a *mom-5* deficit can rescue *unc-40* mutant phase 2 D/V failures, but can only do so in a manner that is strictly dependent on *unc-5* activity. These results not only place *mom-5* and *ced-12* in the same pathway as *unc-5*, they are entirely consistent with a model in which *mom-5* and *ced-12* function upstream of *unc-5* to negatively regulate its function (Fig 10). Furthermore, the effects of *unc-5* and *mom-5* deficits lack reciprocity; *mom-5* deficit does not suppress any *unc-5* mutant DTC migration defects, though *unc-5* deficits suppress *mom-5* mutant DTC migration defects. These results argue against the parallel pathway model and favor a model by which the Wnt receptor MOM-5/Frizzled functions through activated small GTPases to negatively regulate the UNC-5 Netrin receptor (Fig 10).

The above results suggest that *mom-5*, *ced-12*, *ced-10*, and *mig-2* are necessary to negatively regulate *unc-5* in the DTCs during phase 3 migration. However, another question of interest is whether a gain of function in any of these genes is sufficient to negatively regulate *unc-5*. As one example of sufficiency, we found that a *mig-2* gain-of-function mutation causes an *unc-5* mutant-like Unc phenotype as well as DTC phase 2 D/V migration failures. Using a transgene array to drive *unc-5(+)* expression in the DTCs, we were able to rescue the D/V migration defects, supporting the predicted negative regulation of *unc-5* by increased MIG-2 function. We also found that a transgene array *(gly-18p*::*mom-5)* predicted to ectopically express *mom-5* in the DTCs during phase 1 and possibly in the motor neurons was able to suppress an *unc-5* induced precocious dorsal migration during phase 1. This array was also able to induce an *unc-5* mutant-like motor axon D/V guidance defects and corresponding uncoordinated phenotype. This suggests that *mom-5* function, like *mig-2* function, is also sufficient to negatively regulate *unc-5* in the DTCs and possibly in neurons. These gain-of-function results complement the loss-of-function studies suggesting that *unc-5* is a target of negative regulation by *mig-2* and *mom-5* function. These results further support a model by which MOM-5/Frizzled signal through MIG-2/RhoG and CED-10/Rac to negatively regulate *unc-5* during phase 3 migration to prevent DTC phase 3 polarity reversals.

The rescue of the *unc-40* null D/V defects by impairing *mom-5* function demonstrates that *mom-5* is functional during phase 2. The fact that *mom-5* mutations do not normally affect phase 2 DTC migrations (during which *unc-5* expression is already up-regulated), suggests this effect is functionally negligible for phase 2. Instead, *mom-5* becomes functionally relevant for phase 3 to ensure that high levels of *unc-5* function are restricted to phase 2. These effects of MOM-5/Frizzled on UNC-5 function during phase 2 and phase 3 suggest that MOM-5 expression and/or activity levels should begin to increase possibly as early as phase 2 and continue to increase in phase 3 of DTC migration to allow for more robust inhibition of *unc-5*. In full accordance with *mom-5*’s demonstrated role as a negative regulator of *unc-5* function in the execution of phase 2 and in determining the A/P polarity of phase 3 migrations, we found that *mom-5* expression is developmentally up-regulated beginning in phase 2 and further increases in phase 3. This regulation of *mom-5* is temporally correlated, therefore, with its proposed role as a negative regulator of *unc-5*.

We performed several experiments to explore further the mode of regulation imposed by MOM-5/Frizzled on *unc-5*. We examined relative levels of a GFP-tagged UNC-5 translational reporter in the wild type compared to *mom-5(RNAi)* or to *ced-12* null alleles, but observed no obvious changes in GFP abundance or localization within the DTCs in animals compromised for *mom-5* or *ced-12* function. A caveat to these experiments is that the UNC-5::GFP transgenic line overexpresses UNC-5, so a further increase in UNC-5 levels might be masked by this over-expression. However, an increase in UNC-5 activity need not be mediated by an increase in UNC-5 protein expression. There are numerous post-translational means by which MOM-5 could in principle regulate UNC-5’s activity, such as by effects on protein modification like phosphorylation [29,30] or membrane localization. Small GTPases are well known to carry diverse functions. Some of the ascribed functions of small GTPases are to regulate actin dynamics in response to membrane receptors [12,31], however GTPases are also implicated in membrane remodeling and endocytic trafficking of membranal proteins [32–37]. Our use of null alleles places the small GTPases upstream of the UNC-5 receptor and implicate an upstream regulatory role that could potentially align with their proposed function in trafficking. There is some precedence for the latter possibility since both small GTPases and Wnt signals have been found in vertebrates to regulate endocytic recycling of cell adhesion receptors [33–36,38] while in *C*. *elegans* CED-12/ELMO and CED-10/Rac were also found to be involved in endoctyic recycling in the intestinal epithelium [37]. These results raise the possibility that the MOM-5—CED-2,-5,-12—CED-10 signaling pathway, possibly in response to one or more A/P graded external cues, is required to regulate UNC-5 by altering endocytic trafficking of this receptor in the DTCs in a manner that allows normal phase 3 DTC polarity. It should prove interesting to further explore this possibility in the future.

Complex networks of Wnt signaling regulate multiple polarized migrations in *C*. *elegans*. At times, like in the case of the *C*. *elegans* Q neuroblast migration, which migrate in opposite directions with left-right asymmetry, the Wnt signaling pathway that is activated is determined intrinsically [39]. While in other cases a fine balance between various Wnt signaling pathways is crucial for normal patterning [40,41]. We previously found that DTC phase 3 A/P polarity is highly sensitive to the balance between opposing Wnt signaling pathways [2]. Similarly, a balance between various Wnt signals has been found to determine the mirror image division polarities of the vulval precursor cells (VPC) [40], as well as various neuronal migrations [41] suggesting this balance is a common theme in polarity determination in spite of the differences in specific Wnt signals involved in different cell types. It would be interesting to further explore how the *mom-5/unc-5* pathway interplays with other Wnt signaling pathways to determine the A/P polarity of the DTCs and consequently generate the mirror image symmetry of the hermaphrodite gonad.

*unc-5* activity levels seem crucial for the normal manifestation of the different phases of DTC migration. Depending on the context, overexpression as presented here, or loss of function of *unc-5* (in certain Wnt signaling deficient strains) [2], can contribute to A/P polarity reversals. This is very typical of Wnt signaling components. We speculate the UNC-5 might function as a shared module between Wnt signaling and Netrin signaling such that its activity levels relay the readout of both signaling pathways. The regulatory link between MOM-5/Frizzled and UNC-5 presented in this study illuminates how the joint action of Wnt and Netrin signaling is coordinated such that the two signaling pathways manifest an efficient, orchestrated, regulation of polarity formation. We further show that small GTPases can mediate cross talk between these two signaling pathways and propose that small GTPases can function to link other signaling pathways in order to integrate the information from multiple extracellular cues and generate a specific desired outcome in a coordinated manner. The proposed regulatory role of small GTPases presented here also suggests that an outcome of the deregulated activity of small GTPases, which frequently occurs in tumor progression, can consequently result in deregulation of the Netrin receptors. Netrin receptor functions have been implicated in tumorigenic processes such as angiogenesis [42,43], apoptosis, and cell invasion [44–46], thus suggesting UNC-5 or UNC-40/DCC as targets for cancer therapy that might be less deleterious to normal cells than targeting small GTPases. Taken together, the data presented in this study identifies small GTPases as regulatory links allowing for cross talk between Wnt and Netrin signaling pathways, which play central roles in normal development and human disease.

## Materials and Methods

### Nematode culture

Standard procedures were used for the culture, maintenance and genetic analysis of *C*. *elegans* [47]. All strains were grown at 20°C for analysis, unless indicated otherwise. Mutant strains and transgenic lines used in this study are listed in S1 Table. Strains not isolated in our laboratory were obtained from the Caenorhabditis Genetics Center (University of Minnesota), or as indicated in the Acknowledgments section. When necessary, double mutants were verified by PCR; primers are listed in S1 Table.

### Transgenic lines

NW1501 *evIs129* was generated by co-injecting PJJ442 (*emb-9p*::*gfp*, a kind gift from J.M Kramer) and pSU16 *emb-9p*::*unc-5* [4] into N2 and integrated by γ-irradiation. This line was outcrossed 6 times prior to the analysis.

NW2354 *evIs402* was generated by MosSCI single copy insertion into the *oxTi444* locus on LGIII using the EG8080 universal MosSCI insertion strain [48]. Detailed protocol is posted on Wormbuilder web page: http://www.wormbuilder.org/test-page/protocol/.

The plasmid used for the MosSCI insertion (pZH304) was generated by cloning- 2.8kb of the *mom-5* 5’UTR fused to GFP- into the pCFJ350 vector to generate a *mom-5* transcriptional reporter.

The *gly-18p*::*mom-5* transgene was generated by PCR fusion of the *gly-18* promoter amplified from the pAK93-1 (a kind gift from Aldis Krizus) and a *mom-5* genomic fragment amplified from N2 worm lysates. Primers: gly-18A: agtgggcatctttaaaggtagaa;

gly-18A*: gaggatccccatctacaatga;

gly-18_mom-5: cgtatcaagtttttaaataattatttttaaaatttcagATGCATCGACATATTCTGATATTAT

mom-5C: atgcatcgacatattctgatattat; mom-5D: tcataactctaaattcgagacaaag;

mom-5R1:tccgattggctcacattcaca; mom-5R2: gaaatcgagggatgtgctcg

A mix of independently generated PCR fusion products was injected into N2 and VC1848 together with a mix of co-injection markers (P*myo-2*::*mCherry* (pCFJ90); P*myo-3*::*mCherry* (pCFJ104) with or without P*rab-3*::*mChe*rry (pGH8) to generate multiple extrachromosomal array lines (selected lines are listed S1 Table).

### RNA interference (RNAi)

*unc-5(RNAi)* constructs were generated by cloning a 574bp *EcoRI* fragment spanning nucleotides 563–1137 of *unc-5* into the pPD129.36 L4440 vector [49]. In vitro transcribed RNA (Ambion MEGAscript kit) was then injected into young adult hermaphrodites by standard procedures. Bacterial strains for *mom-5(RNAi)* (gene name: T23D8.1 Source Bioscience Location: I-5A13) were obtained from the *C*. *elegans* RNAi library [50]. RNAi by feeding was carried out by standard procedures [51] and compared to the respective control strains grown on the RNAi feeding bacteria HT115(DE3) harboring an empty L4440 vector. In both cases F1 progeny of the RNAi treated worms were analyzed as L4 larvae or adults.

### Generation of synchronized populations

Gravid adults were bleached using 1:5 NaoCl and 0.25N KOH and monitored. Once most embryos were released, the suspension was forced through a 23 gauge needle onto a 45–52μm Nitrex screen. The embryo preparation was then washed 4–5 times with M9 buffer, re-suspended in M9 buffer and incubated overnight at 20°C. Hatched L1 larvae were then plated for further analysis.

### Microscopy

DTC migration patterns were scored by mounting 1mM levamisole-treated animals (L4 or adult stage) on 2% agarose pads for observation using Differential Interference Contrast (DIC) and fluorescence microscopy (Leica DMRA2 or DMRB microscope). Indicated strains carried the *gly-18p*::*gfp* transgene to mark the DTCs. *gly-18p*::*gfp* rarely affects D/V or A/P guidance of the DTC [2]. The A/P polarity reversal phenotype is highly dependent on the incubation temperature. A slight temperature drop below 20°C can result in significantly lower penetrance of the A/P reversals while temperatures above 20°C increase the penetrance of these defects considerably. Care was taken to analyze all comparable strains under the same growth conditions. Therefore, a control strain grown under the same conditions was included in each set of experiments. Data from several independently generated lines were analyzed and the data pooled.

### Quantification of the *mom-5p*::*gfp* signal

Confocal microscopy (C2+ on a Nikon Eclipse NI-E, 60x 1.4NA) was used for the quantification of the *mom5p*::*gfp* signal in order to separate the DTC signal from intestinal *mom-5p*::*gfp* signal. Acquisition parameters were set to ensure that measurements fell within the linear range and the conditions by which the images were acquired were kept constant across samples. The *mom5p*::*gfp* fluorescence intensity in different developmental stages was measured as pixel intensity values sampling a fixed size circle drawn around the nucleus of the DTCs in a single plane which was chosen using DIC optics through the axial center of the nucleus.

### Statistical analysis

Standard errors of the proportion (SE) were calculated assuming a binomial distribution of the observed proportion and the actual sample size. Statistical tests were carried out using a standard (two-tailed) comparison of two proportions (Z test). All P values represent the probability that the measured frequency of the phenotype is the same for the two strains being compared. A P-value of less than 0.01 is considered significant. All comparisons described as significant in the Results section were based on this criterion.

## Supporting Information

S1 Fig *mom-5(gk812)* is null for the DTC phase 3 A/P polarity phenotype and the *gly-18p*::*mom-5* transgene rescues the *mom-5(gk812)* DTC A/P polarity reversals. (A) Red (anterior DTCs) and black (posterior DTCs) bars represent the percentage of phase 3 polarity reversals in *mom-5(RNAi)* treated animals and *mom-5(gk812)* maternally rescued homozygotes treated or not with *mom-5(RNAi)*. The corresponding raw data are presented in S8 Table. Error bars indicate standard error of the sample proportion. ns = not significant (P≥0.01). *mom-5(RNAi)* was effected by feeding animals balanced for the maternal effect lethal *gk812* allele. Controls were grown on empty vector feeding bacteria. The experiment was carried out at 25°C to maximize the efficacy of the RNAi. (B) Quantification of phase 3 A/P polarity reversals in *mom-5(gk812)* maternally rescued homozygotes and *mom-5(gk812)* homozygotes carrying the *gly-18p*::*mom-5(+)* array. Bars represent the percentage of phase 3 polarity reversals for anterior DTCs (red bars) and for posterior DTCs (black bars). Error bars indicate standard error of the sample proportion. ***P <0.00001.
(EPS)

S2 Fig A/P polarity reversals induced by *evIs129[emb-9p*::*unc-5]* are enhanced by *mom-5(RNAi)* and *ced-12(k149)*. Bars represent the percentage of phase 3 polarity reversals for anterior DTCs (red bars) and for posterior DTCs (black bars). The corresponding raw data are presented in S9 Table. Error bars indicate standard error of the sample proportion. *P<0.01; **P<0.001; ns = not significant (P≥0.01). *mom-5(RNAi)* was effected by feeding animals balanced for the maternal effect lethal *gk812* allele. Controls for RNAi were grown on empty vector feeding bacteria. A slight temperature drop below 20 degrees can result in significantly lower penetrance of the A/P reversals while temperatures above 20 degrees increase the penetrance of these defects considerably. Care was taken to analyze all comparable strains under the same growth conditions; therefore, a control strain grown under the same conditions was included in each set of experiments.
(EPS)

S3 Fig A MIG-2/RhoG protein fusion reporter is expressed in the hermaphrodite DTCs throughout development. Fluorescence photomicrographs of hermaphrodites carrying *muIs27*, an integrated *mig-2p*::*mig-2*::*gfp* transgene array [22]. Anterior is left and dorsal is up. Arrows mark the DTC. Asterisk marks the vulva. Developmental stage is indicated on the right. L1-L4 represent larval stages preceding the adult stage. In L1 and the adult stages, both DTCs are shown. In L2 and L4 only the posterior DTC is shown.
(EPS)

S4 Fig *gly-18* is expressed in the DTCs at the onset of the first migratory phase and in the ventral nerve cord. DIC (A) and fluorescence photomicrographs (A’) and an overlay of the two (A”) of L1/L2 hermaphrodites carrying *dnIs13[gly-18p*::*gfp]*, an integrated *gly-18* transcriptional transgenic reporter array. Anterior is left and dorsal is up. Arrows mark the DTCs. (B) Fluorescence photomicrographs of adult hermaphrodite carrying *dnIs13[gly-18p*::*gfp]*. Anterior is left and dorsal is up.
(EPS)

S5 Fig Ectopic expression of *mom-5* during phase 1 is sufficient to suppress the *emb-9p*::*unc-5* induced precocious dorsal migration. Bars represent the percentage of DTCs that migrate precociously to the dorsal side in synchronized populations analyzed 24 hours after plating of L1 larva grown at 25°C. Red fluorescence, RFP(+), indicates the presence of the *gly-18p*::*mom-5* extrachromosomal transgenic array that was created by co-injecting a mix of mCherry markers (S1 Table). These segregated some RFP(-) hermaphrodites that lacked the *gly-18p*::*mom-5* array, which served as the *evIs129* control. Error bars indicate standard error of the sample proportion. ***P <0.00001; ns = not significant (P≥0.01).
(EPS)

S6 Fig *mom-5*::*gfp* is undetectable in the DTCs throughout phase 1, while *unc-5*::*gfp* is up regulated at the end of phase 1 prior to the first turn. Upper panels: DIC and fluorescence micrographs of hermaphrodites carrying an integrated *mom-5p*::*gfp* single copy insertion (*evIs462*). Anterior is left and dorsal is up. Arrows mark the DTC. Sequential stages of DTC migration are shown starting at the L1 stage to the L3 to L4 stage (end of phase 1). Except for L1 and L2 only a single gonad arm is shown. For *evIs462* no GFP is visible in the DTCs (gain and exposure times are identical to those of images presented in Fig 9). Lower panels: DIC and fluorescence micrographs of hermaphrodites carrying an integrated *unc-5p*::*unc-5gfp* transgene array (*evIs98C*). DTC GFP expression (arrows) is first visible just before or during the first DTC turn.
(EPS)

S7 Fig *mom-5* is transcriptionally activated prior to the onset of phase 3. The average pixel intensity of *mom-5p*::*gfp* expression in the DTC is represented in several stages throughout DTC migration. The corresponding one way ANOVA column statistics data are presented in S7 Table.
(EPS)

S1 Table Strains used in the analysis. (DOCX)

S2 Table A/P polarity reversals and D/V precocious migration defects in *evIs98C[unc-5p*::*unc-5*::*gfp]* or *evIs129[emb-9p*::*unc-5]* in the wild type or in the background of Netrin signaling components mutants^1^. ^1^DTC migration patterns were analyzed by DIC and fluorescence optics of anterior and posterior DTCs in L4 larvae or adults. ***P<0.00001; ^ns^P≥0.01 n = number of gonad arms scored (anterior and posterior combined). SE = standard error of the proportion. ^2^The penetrance of defects in the *evIs129* transgenic line was highly temperature sensitive and variable. Care was taken to analyze the respective control for each experiment grown under the same conditions. ^3^Grown on empty vector RNAi feeding bacteria.
(DOCX)

S3 Table A/P polarity reversals or D/V migration defects in *mom-5* mutant alleles treated or not with *unc-5(RNAi)* and Netrin receptor mutants treated or not with *mom-5(RNAi)* ^1^. ^1^DTC migration patterns were analyzed for anterior and posterior DTCs by DIC and fluorescent optics in L4 larvae or adults. ***P<0.00001; **P<0.001; *P<0.01; ^ns^P≥0.01. n = number of gonad arms scored. SE = standard error of the proportion. ^2^ *mom-5(gk812)* and *mom-5(zu193)* are maternal effect embryonic lethal mutations. Balanced heterozygotes were injected with *unc-5(RNAi)* and the mutant homozygous progeny were analyzed for DTC migration defects; *gk812* were identified as non-*gfp*, *zu193* identified as Unc (S1 Table). ^3^ *unc-5(RNAi)* was introduced by ds RNA injection into the different strains, the D/V migration defects in the *unc-5(RNAi)* set of experiments reflect the efficacy of the RNAi treatment, which due to the method of delivery can be variable from strain to strain. ^4^ *mom5(RNAi)* was administered by feeding to bypass the associated embryonic lethality. This also allowed comparison of the RNAi induced defect across all strains, all grown on the same RNAi feeding bacteria under the same growth conditions.
(DOCX)

S4 Table A/P polarity reversals or D/V migration defects in *ced-12* mutant alleles alone or in the background of Netrin signaling components mutants^1^. ^1^DTC migration patterns for anterior and posterior DTCs were analyzed by DIC and fluorescence optics in L4 larvae or adults. ***P<0.00001; **P<0.001; *P<0.01; ^ns^P≥0.01. n = number of gonad arms scored. SE = standard error of the proportion. The A/P polarity reversal phenotype is highly temperature sensitive. Care was taken to analyze a respective control grown under the same incubation conditions for each set of experiments.
(DOCX)

S5 Table A/P polarity reversals in *ced-10* mutant alleles treated or not with *unc-5(RNAi)* ^1^. ^1^DTC migration patterns of anterior and posterior DTCs were analyzed by DIC and florescence optics in L4 larvae or adults. ***P<0.00001; *P<0.01; ^ns^P≥0.01. n = number of gonad arms scored. SE = standard error of the proportion. ^2^D/V guidance defects result from impairing *unc-5* function and reflect the efficacy of the *unc-5(RNAi)* in the population. ^2^ *ced-10(n3417)* is a maternal effect embryonic lethal mutation. Balanced heterozygotes were injected with *unc-5(RNAi)* and the *ced-10(n3417)* homozygous progeny (identified as non-dpy) were analyzed for DTC migration defects. ^3^ *ced-10(t1875)* is a maternal effect embryonic lethal mutation. Balanced heterozygotes were injected with *unc-5(RNAi)* and the *ced-10(t1875)* homozygous progeny identified as GFP(-) were analyzed for DTC migration defects.
(DOCX)

S6 Table A/P polarity reversals or D/V migration defects in *mig-2* null alleles alone or in the background of *unc-5* mutants or *unc-5(RNAi)* as well as DTC migration defects of *mig-2(gm103)* in the presence or absence of *evIs129* ^1^. ^1^DTC migration patterns of anterior and posterior DTC were analyzed by DIC and florescence optics in L4 larvae or adults. n = number of gonad arms scored. SE = standard error of the proportion. ***P<0.00001; **P<0.001; *P<0.01; ^ns^P≥0.01. ^2^It should be noted that we refrained from comparing the penetrance of D/V migration failures when *unc-5(RNAi)* was used. *unc-5(RNAi)* was delivered by injection; the efficacy of the RNAi treatment can vary between the injected strains. The percent D/V defects in the *unc-5(RNAi)* set of experiments reflect the efficacy of the RNAi. ^3^ *mig-2(gm103) evIs129/+* were used. Only the *emb-9p*::*gfp* positive animals were included in the analysis; these may have been either heterozygous or homozygous for *evIs129*.
(DOCX)

S7 Table *mom-5* is transcriptionally activated prior to the onset of phase 3. One way ANOVA column statistics.
(DOCX)

S8 Table *mom-5(gk812)* A/P polarity reversals are not enhanced by *mom-5(RNAi)* ^*1*^. ^1^DTC migration patterns of anterior and posterior DTC were analyzed by DIC in L4 larvae or adults. n = number of gonad arms scored. SE = standard error of the proportion. ^ns^P≥0.01. ^2^ *mom-5(gk812)* is a maternal effect embryonic lethal mutation. Balanced heterozygotes were fed with *mom-5(RNAi)* and the *mom-5* mutant homozygous progeny were analyzed for DTC migration defects; the balancer chromosome is marked with gfp hence *gk812* homozygotes were identified as non-*gfp* (S1 Table).
(DOCX)

S9 Table *evIs129* A/P polarity reversals are enhanced by *mom-5(gk812) and ced-12(k149)* ^*1*^. ^1^DTC migration patterns of anterior and posterior DTC were analyzed by DIC in L4 larvae or adults. n = number of gonad arms scored. SE = standard error of the proportion. **P<0.001; *P<0.01; ^ns^P≥0.01 ^2^RNAi was introduced by feeding; controls were grown on empty vector feeding bacteria. ^3^The penetrance of defects in the *evIs129* transgenic line was highly temperature sensitive and variable. Care was taken to analyze the respective control for each experiment grown under the same conditions.
(DOCX)
