# Supplementary material for: The Wnt Frizzled Receptor MOM-5 Regulates the UNC-5 Netrin Receptor through Small GTPase-Dependent Signaling to Determine the Polarity of Migrating Cells
Source: PLoS Genet. 2015 Aug 20;11(8):e1005446. doi: 10.1371/journal.pgen.1005446 (PMC4546399; doi:10.1371/journal.pgen.1005446)
Supplement: S1 Table — (DOCX) [file pgen.1005446.s008.docx]

**S1 Table. Strains used in the analysis**

| **Strain** | ***C. elegans* gene** | **Encoded product** | **Allelic description** |
| --- | --- | --- | --- |
| **DE60** | unc-119(e2498); dnIs13*[gly-18p::gfp; unc-119(+)]* | GFP reporter for DTCs | *gly-18p::gfp* is expressed in the DTCs [1]  Kindly provided by Wendy Johnston and Aldis Krizus |
| **NW2256** | *unc-6(ev400);* dnIs13 | Netrin (-) | Putative null [2] |
| **NW2253** | *unc-40(e1430);* dnIs13 | Netrin receptor (DCC) (-) | Putative null [2] |
| **NW2252** | *unc-5(e53);* dnIs13 | Netrin receptor (UNC-5) (-) | Putative null W283 TAG [3] |
| **NW2254** | *unc-5(ev489);* dnIs13 | Netrin receptor (UNC-5) (-) | Putative null W41 [3] |
| **NW1166** | *evIs98C[unc-5p::unc-5::gfp; dpy-20(+)]; dpy-20(e1232)* | Netrin receptor (UNC-5) (+++) | Transgenic line: *unc-5* translational reporter, GFP fusion protein [4] |
| **NW1501** | *evIs129[emb-9p::unc-5; emb-9p:: gfp]; him-5* | Netrin receptor (UNC-5) (+++) | Transgenic line: *unc-5 or* GFP driven by the *emb-9* promoter, integrated on LGX |
| **NW2339** | *unc-40(e1430); evIs129[emb-9p::unc-5; emb-9p::gfp]* | DCC (-); UNC-5 (+++) |  |
| **NW2340** | *evIs129[emb-9p::unc-5; emb-9p::gfp] unc-6(ev400)* | Netrin (-) UNC-5 (+++) |  |
| **NW2371** | *ced-12(k149);* dnIs13 | ELMO (-), GEF | Point mutation R38>STOP, probably a null [5] |
| **NW2341** | *ced-12(k149); unc-5(e53);* dnIs13 | ELMO (-); UNC-5 (-) |  |
| **NW2342** | *ced-12(k149); unc-5(ev489);* dnIs13 | ELMO (-); UNC-5 (-) |  |
| **NW2343** | *unc-40(e1430) ced-12(k149);* dnIs13 | UNC-40 (-) ELMO (-) |  |
| **NW2344** | *ced-12(k149); unc-6(ev400);* dnIs13 | ELMO (-); Netrin (-) |  |
| **NW2372** | *ced-12(n3261);* dnIs13 | ELMO (-), GEF | R262 > opal stop [6] |
| **NW2345** | *ced-12(n3261); unc-5(e53);* dnIs13 | ELMO (-); UNC-5 (-) |  |
| **NW2346** | *ced-12(n3261); unc-5(ev489);* dnIs13 | ELMO (-); UNC-5 (-) |  |
| **NW2347** | *unc-40(e1430) ced-12(n3261);* dnIs13 | UNC-40 (-) ELMO (-) |  |
| **MT10869** | *ced-10(n3417)/lin-1(e1275) dpy-13(e184)* | Rac (-) | *ced-10(n3417)* lacks exons two and three and is predicted to result in the expression of only the first 35 amino acids of CED-10. [7] |
| **WS2384** | *ced-10(t1875)/nT1IVandVqIs51IV;V* | Rac (-) | Results in a lesion that alters the initial ATG. Putative null [8] |
| **NW2373** | *mig-2(mu28);* dnIs13 | Rho G (-) | W60 > opal stop putative null [9] |
| **NW2348** | *unc-5(ev489); mig-2(mu28);* dnIs13 | UNC-5 (-); Rho G (-) |  |
| **NW2349** | *unc-5(e53); mig-2(mu28);* dnIs13 | UNC-5 (-); Rho G (-) |  |
| **VC1848** | *mom-5(gk812) I/hT2 [bli-4(e937) let-?(q782) qIs48] (I;III).* | Wnt Frizzled receptor (-) | Insertion/deletion Insertion: GAAAAAGTTTGG (mutant) Deletion: 567 bp deletion. Putative null (S1 Fig) |
| **EU452** | *mom-5(zu193) unc-13(e1091)/hT2 I; +/hT2 [bli-4(e937) let-?(h661)] III.* | Wnt Frizzled receptor (-) | Putative null [10] |
| **NW2354** | *evIs462[mom-5p::gfp;unc-119(+)]; unc-119(ed3)* | *mom-5* transcriptional reporter | MosSCI single copy insertion oxTi444 locus on LGIII |
| **NW2351** | *evEx457[unc-5p::gfp]* | *unc-5* transcriptional reporter |  |
| **CF579** | *dpy-20(e1282) IV; him-5(e1490) V; muIs27* | *mig-2* translational reporter | Transgenic line: *mig-2* translational reporter, GFP fusion protein [9] |
| **NW2380** | *mig-2(gm103) evIs129/+* | RhoG(gof); UNC-5(++) | *mig-2(gm103)* homozygote; *evIs129* heterozygote |
| **NW2381** | *mig-2(gm103) evIs129* | RhoG(gof); UNC-5(+++) |  |
| **NW2382-**  **NW2384** | *evEx464/465/466[gly-18p::mom-5; myo-3p::mCherry; myo-2p::mCherry; rab-3p::mCherry]* | Wnt Frizzled receptor (+++) | Wnt Frizzled receptor driven by the *gly-18* promoter in N2 |
| **NW2385-**  **NW2389** | *mom-5(gk812) I/hT2 [bli-4(e937) let-?(q782) qIs48] (I;III);evEx467/468/469/470/471[gly-18p::mom-5; myo-3p::mCherry; myo-2p::mCherry; rab-3p::mCherry]* | Wnt Frizzled receptor (+++) | Wnt Frizzled receptor driven by the *gly-18* promoter in *mom-5(gk812)* |
| **NW2390-**  **NW2392** | *evEx472/473/474[gly-18p::mom-5; myo-3p::mCherry; myo-2p::mCherry]* | Wnt Frizzled receptor (+++) | Wnt Frizzled receptor driven by the *gly-18* promoter in N2 |
| **NW2397** | *ced-12(k149);evIs129* | ELMO (-); UNC-5 (+++) |  |
| **NW1100** | *evIs82[*unc-129::gfp; dpy-20(+)] | A member of the TGF-β superfamily | unc-129::gfp is expressed in the DA and DB motor neurons |
| **NW2404** | *evEx466;evIs82[*unc-129*::gfp; dpy-20(+)]* | Wnt Frizzled receptor (+++) |  |
| **NW2393** | *evEx475[gly-18p::mom-5; myo-3p::mCherry; myo-2p::mCherry] in evIs129* | Wnt Frizzled receptor (+++); UNC-5(+++) | Wnt Frizzled receptor driven by the *gly-18* promoter in *evIs129* |

**Genotyping primers**

| **Strain** | **Forward primer** | **Reverse primer** | **Enzymatic digest** |
| --- | --- | --- | --- |
| *ced-12(k149)* | attgatcgagctgccattttcacgaca | cgctagatttttcaagagaggttggttaccttc | TaqI |
| *ced-12(n3261) WT* | gaacaccaccaccaccacgtagaagagaa*c* | cattcgacacgttgaaaaatcagatgaac |  |
| *ced-12(n3261)* mutant | gaacaccaccaccaccacgtagaagagaa*c* | cattcgacacgttgaaaaatcagatgaat |  |
| *unc-40(e1430)* | ggatagaatcagtgagcagtgccg | gacatgggcttggagcctca | DpnII |
| *unc-5(e53)* | gacgagaagctgtaaagtaccatgtaaacttgatggaggatggagtccatggagtgat | ctcactgtgccggacactcttgtgtc | XcmI |
| *mig-2(mu28)* | ggaaagtggtcgtaaacgatcataatcctcctgtccagcaagatc | gtagttgttggagacggaacag | BglII |
| *mig-2(gm103)* WT | catgacagtattcgggtgccagc | catgtttttccaactgttccgtctc |  |
| *mig-2(gm103) mutant* | catgacagtattcgggtgccagc | catgcatgtttttccaactgtttc |  |
| *evIs98C* | gtttgtgtagagttccgattgcc | acaccgatggaccagaatcaac |  |

**References**

1. Warren CE, Krizus a, Dennis JW (2001) Complementary expression patterns of six nonessential Caenorhabditis elegans core 2/I N-acetylglucosaminyltransferase homologues. Glycobiology 11: 979–988. doi:10.1093/glycob/11.11.979.

2. Hedgecock EM, Culotti JG, Hall DH (1990) The unc-5, unc-6, and unc-40 genes guide circumferential migrations of pioneer axons and mesodermal cells on the epidermis in C. elegans. Neuron 4: 61–85.

3. Killeen M, Tong J, Krizus A, Steven R, Scott I, et al. (2002) UNC-5 Function Requires Phosphorylation of Cytoplasmic Tyrosine 482, but Its UNC-40-Independent Functions also Require a Region between the ZU-5 and Death Domains. Dev Biol 251: 348–366. doi:10.1006/dbio.2002.0825.

4. Su M, Merz DC, Killeen MT, Zhou Y, Zheng H, et al. (2000) Regulation of the UNC-5 netrin receptor initiates the first reorientation of migrating distal tip cells in Caenorhabditis elegans. Development 127: 585–594.

5. Nishiwaki K (1999) Mutations affecting symmetrical migration of distal tip cells in Caenorhabditis elegans. Genetics 152: 985–997.

6. Zhou Z, Caron E, Hartwieg E, Hall a, Horvitz HR (2001) The C. elegans PH domain protein CED-12 regulates cytoskeletal reorganization via a Rho/Rac GTPase signaling pathway. Dev Cell 1: 477–489.

7. Lundquist E a, Reddien PW, Hartwieg E, Horvitz HR, Bargmann CI (2001) Three C. elegans Rac proteins and several alternative Rac regulators control axon guidance, cell migration and apoptotic cell phagocytosis. Development 128: 4475–4488.

8. Kinchen JM, Cabello J, Klingele D, Wong K, Feichtinger R, et al. (2005) Two pathways converge at CED-10 to mediate actin rearrangement and corpse removal in C. elegans. Nature 434: 93–99. doi:10.1038/nature03263.

9. Zipkin ID, Kindt RM, Kenyon CJ (1997) Role of a new Rho family member in cell migration and axon guidance in C. elegans. Cell 90: 883–894.

10. Cabello J, Neukomm LJ, Günesdogan U, Burkart K, Charette SJ, et al. (2010) The Wnt pathway controls cell death engulfment, spindle orientation, and migration through CED-10/Rac. PLoS Biol 8: e1000297. doi:10.1371/journal.pbio.1000297.
