# Supplementary material for: The Wnt Frizzled Receptor MOM-5 Regulates the UNC-5 Netrin Receptor through Small GTPase-Dependent Signaling to Determine the Polarity of Migrating Cells
Source: PLoS Genet. 2015 Aug 20;11(8):e1005446. doi: 10.1371/journal.pgen.1005446 (PMC4546399; doi:10.1371/journal.pgen.1005446)
Supplement: S2 Table — 1DTC migration patterns were analyzed by DIC and fluorescence optics of anterior and posterior DTCs in L4 larvae or adults. ***P<0.00001; nsP≥0.01 n = number of gonad arms scored (anterior and posterior combined). SE = standard error of the proportion. 2The penetrance of defects in the evIs129 transgenic line was highly temperature sensitive and variable. Care was taken to analyze the respective control for each experiment grown under the same conditions. 3Grown on empty vector RNAi feeding bacteria. (DOCX) [file pgen.1005446.s009.docx]

**S2 Table. A/P polarity reversals and D/V precocious migration defects in *evIs98C[unc-5p::unc-5::gfp]* or *evIs129[emb-9p::unc-5]* in the wild type or in the background of Netrin signaling components mutants^1^**

|  | **A/P polarity reversals** | | | **D/V precocious migration** | | |
| --- | --- | --- | --- | --- | --- | --- |
| **Strain** | **%** | **SE** | **n** | **%** | **SE** | **n** |
| *evIs129[emb-9p::unc-5; emb-9p::gfp]^2,3^* | 34 | 2 | 490 | 30 | 2 | 490 |
| *unc-5(RNAi); evIs129[emb-9p::unc-5; emb-9p::gfp]* | 9^***^ | 1 | 664 | 1^***^ | 0.5 | 664 |
| *evIs98C**[unc-5p::unc-5::gfp]* | 66 | 4 | 154 | 0 | 0 | 154 |
| *evIs129[emb-9p::unc-5; emb-9p::gfp]^2^* | 54 | 3 | 265 | 68 | 3 | 265 |
| *unc-40(e1430); evIs129[emb-9p::unc-5; emb-9p::gfp]* | 15^***^ | 2 | 247 | 40^***^ | 3 | 246 |
| *evIs129[emb-9p::unc-5; emb-9p::gfp]^2^* | 51 | 3 | 330 | 64 | 3 | 330 |
| *evIs129[emb-9p::unc-5; emb-9p::gfp] unc-6(ev400)* | 48^ns^ | 4 | 147 | 3^***^ | 1 | 147 |

^1^DTC migration patterns were analyzed by DIC and fluorescence optics of anterior and posterior DTCs in L4 larvae or adults. ^***^P<0.00001; ^ns^P≥0.01

n = number of gonad arms scored (anterior and posterior combined). SE = standard error of the proportion.

^2^The penetrance of defects in the *evIs129* transgenic line was highly temperature sensitive and variable. Care was taken to analyze the respective control for each experiment grown under the same conditions.

^3^Grown on empty vector RNAi feeding bacteria.
