# Supplementary material for: The Wnt Frizzled Receptor MOM-5 Regulates the UNC-5 Netrin Receptor through Small GTPase-Dependent Signaling to Determine the Polarity of Migrating Cells
Source: PLoS Genet. 2015 Aug 20;11(8):e1005446. doi: 10.1371/journal.pgen.1005446 (PMC4546399; doi:10.1371/journal.pgen.1005446)
Supplement: S3 Table — 1DTC migration patterns were analyzed for anterior and posterior DTCs by DIC and fluorescent optics in L4 larvae or adults. ***P<0.00001; **P<0.001; *P<0.01; nsP≥0.01. n = number of gonad arms scored. SE = standard error of the proportion. 2 mom-5(gk812) and mom-5(zu193) are maternal effect embryonic lethal mutations. Balanced heterozygotes were injected with unc-5(RNAi) and the mutant homozygous progeny were analyzed for DTC migration defects; gk812 were identified as non-gfp, zu193 identified as Unc (S1 Table). 3 unc-5(RNAi) was introduced by ds RNA injection into the different strains, the D/V migration defects in the unc-5(RNAi) set of experiments reflect the efficacy of the RNAi treatment, which due to the method of delivery can be variable from strain to strain. 4 mom5(RNAi) was administered by feeding to bypass the associated embryonic lethality. This also allowed comparison of the RNAi induced defect across all strains, all grown on the same RNAi feeding bacteria under the same growth conditions. (DOCX) [file pgen.1005446.s010.docx]

**S3 Table. A/P polarity reversals or D/V migration defects in *mom-5* mutant alleles treated or not with *unc-5(RNAi)* and Netrin receptor mutants treated or not with *mom-5(RNAi)*^1^**

|  | **A/P polarity reversals** | | | | | | **D/V migration failures** | | | | | |
| --- | --- | --- | --- | --- | --- | --- | --- | --- | --- | --- | --- | --- |
|  | **Anterior** | | | **Posterior** | | | **Anterior** | | | **Posterior** | | |
| **Strain** | **%** | **SE** | **n** | **%** | **SE** | **n** | **%** | **SE** | **n** | **%** | **SE** | **n** |
| *mom-5(gk812)^2^* | 56 | 5 | 98 | 84 | 4 | 97 | 0 | 0 | 98 | 0 | 0 | 97 |
| *mom-5(gk812); unc-5(RNAi)* | 35^*^ | 5 | 100 | 26^***^ | 4 | 100 | 6 | 2 | 100 | 25 | 4 | 100 |
| *unc-5(RNAi)^3^* | 0 | 0 | 87 | 0 | 0 | 87 | 15 | 4 | 87 | 47 | 5 | 87 |
| *mom-5(zu193)^2^* | 74 | 4 | 103 | 65 | 5 | 104 | 0 | 0 | 103 | 0 | 0 | 104 |
| *mom-5(zu193); unc-5(RNAi)* | 44^**^ | 7 | 52 | 19^***^ | 5 | 52 | 21 | 6 | 52 | 29 | 6 | 52 |
| *mom-5(RNAi)* | 39 | 4 | 191 | 26 | 3 | 191 | 0 | 0 | 191 | 1 | 0 | 191 |
| *mom-5(RNAi); unc-5(e53)* | 38 ^ns^ | 4 | 159 | 9^***^ | 2 | 158 | 32 ^ns^ | 4 | 159 | 71 ^ns^ | 4 | 158 |
| *unc-5(e53)* | 3 | 1 | 161 | 1 | 1 | 161 | 35 | 4 | 161 | 69 | 4 | 161 |
| *mom-5(RNAi); unc-5(ev489)* | 30^ns^ | 5 | 83 | 6^**^ | 3 | 83 | 34 ^ns^ | 5 | 83 | 68 ^ns^ | 5 | 83 |
| *unc-5(ev489)* | 3 | 1 | 260 | 4 | 1 | 260 | 29 | 3 | 260 | 71 | 3 | 260 |
| *mom-5(RNAi)^4^* | 44 | 4 | 163 | 53 | 4 | 163 | 1 | 1 | 163 | 1 | 1 | 163 |
| *unc-40(e1430) mom-5(RNAi)* | 13^***^ | 3 | 121 | 3^***^ | 2 | 121 | 8 ^ns^ | 3 | 121 | 10^***^ | 3 | 121 |
| *unc-40(e1430)* | 0 | 0 | 107 | 0 | 0 | 107 | 5 | 2 | 107 | 36 | 5 | 107 |
| *unc-40(e1430); evIs129[emb-9p::unc-5)* |  |  |  |  |  |  | 1 ^ns^ | 1 | 123 | 6^***^ | 2 | 122 |
| *mom-5(RNAi)* | 59 | 5 | 96 | 49 | 5 | 96 | 0 | 0 | 96 | 0 | 0 | 96 |
| *unc-5(e53)* | 3 | 1 | 155 | 0 | 0 | 155 |  |  |  |  |  |  |
| *mom-5(RNAi); unc-5(e53)* | 27^***^ | 4 | 112 | 16^***^ | 1 | 112 |  |  |  |  |  |  |
| *unc-40(e1430); unc-5(e53)* | 2 | 1 | 186 | 1 | 1 | 186 | 34 | 3 | 186 | 80 | 3 | 186 |
| *unc-40(e1430) mom-5(RNAi); unc-5(e53)* | 22^***^ | 4 | 130 | 8^***^ | 1 | 131 | 57^*^ | 2 | 129 | 81 ^ns^ | 1 | 130 |

^1^DTC migration patterns were analyzed for anterior and posterior DTCs by DIC and fluorescent optics in L4 larvae or adults. ^***^P<0.00001; ^**^P<0.001; ^*^P<0.01; ^ns^P≥0.01.

n = number of gonad arms scored. SE = standard error of the proportion.

^2^*mom-5(gk812)* and *mom-5(zu193)* are maternal effect embryonic lethal mutations. Balanced heterozygotes were injected with *unc-5(RNAi)* and the mutant homozygous progeny were analyzed for DTC migration defects; *gk812* were identified as non-*gfp*, *zu193* identified as Unc (S1 Table).

^3^ *unc-5(RNAi)* was introduced by ds RNA injection into the different strains, the D/V migration defects in the *unc-5(RNAi)* set of experiments reflect the efficacy of the RNAi treatment, which due to the method of delivery can be variable from strain to strain.

^4^*mom5(RNAi)* was administered by feeding to bypass the associated embryonic lethality. This also allowed comparison of the RNAi induced defect across all strains, all grown on the same RNAi feeding bacteria under the same growth conditions.
