# Supplementary material for: The Wnt Frizzled Receptor MOM-5 Regulates the UNC-5 Netrin Receptor through Small GTPase-Dependent Signaling to Determine the Polarity of Migrating Cells
Source: PLoS Genet. 2015 Aug 20;11(8):e1005446. doi: 10.1371/journal.pgen.1005446 (PMC4546399; doi:10.1371/journal.pgen.1005446)
Supplement: S4 Table — 1DTC migration patterns for anterior and posterior DTCs were analyzed by DIC and fluorescence optics in L4 larvae or adults. ***P<0.00001; **P<0.001; *P<0.01; nsP≥0.01. n = number of gonad arms scored. SE = standard error of the proportion. The A/P polarity reversal phenotype is highly temperature sensitive. Care was taken to analyze a respective control grown under the same incubation conditions for each set of experiments. (DOCX) [file pgen.1005446.s011.docx]

**S4 Table. A/P polarity reversals or D/V migration defects in *ced-12* mutant alleles alone or in the background of Netrin signaling components mutants^1^**

|  | **A/P polarity reversals** | | | | | | **D/V migration failures** | | | | | |
| --- | --- | --- | --- | --- | --- | --- | --- | --- | --- | --- | --- | --- |
|  | **Anterior** | | | **Posterior** | | | **Anterior** | | | **Posterior** | | |
| **Strain** | **%** | **SE** | **n** | **%** | **SE** | **n** | **%** | **SE** | **n** | **%** | **SE** | **n** |
| *ced-12(k149);* *dnIs13* | 18 | 1 | 1364 | 25 | 1 | 1360 | 0.5 | 0 | 1364 | 1 | 0.5 | 1360 |
| *unc-5(e53);* *dnIs13* | 3 | 1 | 161 | 1 | 1 | 161 | 35 | 4 | 161 | 69 | 4 | 161 |
| *ced-12(k149); unc-5(e53);* *dnIs13* | 9^**^ | 2 | 230 | 8^***^ | 2 | 236 | 45 ^ns^ | 3 | 230 | 69 ^ns^ | 3 | 236 |
| *unc-5(ev489);* *dnIs13* | 3 | 1 | 260 | 0.4 | 0.4 | 260 | 29 | 3 | 260 | 71 | 3 | 260 |
| *ced-12(k149); unc-5(ev489);* *dnIs13* | 13^*^ | 2 | 318 | 10^***^ | 2 | 322 | 48 ^***^ | 3 | 318 | 61 ^ns^ | 3 | 322 |
| *ced-12(k149); unc-5(RNAi);* *dnIs13* | 8^**^ | 1 | 471 | 11^***^ | 1 | 472 | 25 | 2 | 471 | 29 | 2 | 472 |
| *unc-40(e1430);* *dnIs13* | 1 | 1 | 94 | 3 | 2 | 94 | 15 | 4 | 94 | 45 | 5 | 94 |
| *unc-40(e1430) ced-12(k149);* *dnIs13* | 7^***^ | 1 | 363 | 8^***^ | 1 | 360 | 26 ^ns^ | 2 | 363 | 38^ns^ | 3 | 360 |
| *unc-6(ev400);* *dnIs13* | 3 | 1 | 151 | 1 | 1 | 151 | 44 | 4 | 151 | 70 | 4 | 151 |
| *ced-12(k149); unc-6(ev400);* *dnIs13* | 25^*^ | 2 | 340 | 22 ^ns^ | 2 | 358 | 50 ^ns^ | 3 | 340 | 82^*^ | 2 | 358 |
| *ced-12(n3261);* *dnIs13* | 16 | 2 | 516 | 36 | 2 | 516 | 1 | 0.5 | 516 | 1 | 0.5 | 516 |
| *unc-5(e53);* *dnIs13* | 3 | 1 | 161 | 1 | 1 | 161 | 35 | 4 | 161 | 69 | 4 | 161 |
| *ced-12(n3261); unc-5(e53);* *dnIs13* | 13^ns^ | 3 | 129 | 11^***^ | 3 | 132 | 35 ^ns^ | 4 | 129 | 61 ^ns^ | 4 | 132 |
| *unc-5(ev489);* *dnIs13* | 3 | 1 | 260 | 0.4 | 0.4 | 260 | 29 | 3 | 260 | 71 | 3 | 260 |
| *ced-12(n3261); unc-5(ev489);* *dnIs13* | 6^**^ | 2 | 115 | 9^***^ | 3 | 115 | 51^***^ | 5 | 115 | 72 ^ns^ | 4 | 115 |
| *unc-40(e1430);* *dnIs13* | 1 | 1 | 94 | 3 | 2 | 94 | 15 | 4 | 94 | 45 | 5 | 94 |
| *unc-40(e1430) ced-12(n3261);* *dnIs13* | 4^***^ | 1 | 193 | 7^***^ | 2 | 193 | 36^**^ | 4 | 193 | 25^**^ | 3 | 193 |

^1^DTC migration patterns for anterior and posterior DTCs were analyzed by DIC and fluorescence optics in L4 larvae or adults.

^***^P<0.00001; ^**^P<0.001; ^*^P<0.01; ^ns^P≥0.01.

n = number of gonad arms scored. SE = standard error of the proportion. The A/P polarity reversal phenotype is highly temperature sensitive. Care was taken to analyze a respective control grown under the same incubation conditions for each set of experiments.
