# Supplementary material for: The Wnt Frizzled Receptor MOM-5 Regulates the UNC-5 Netrin Receptor through Small GTPase-Dependent Signaling to Determine the Polarity of Migrating Cells
Source: PLoS Genet. 2015 Aug 20;11(8):e1005446. doi: 10.1371/journal.pgen.1005446 (PMC4546399; doi:10.1371/journal.pgen.1005446)
Supplement: S5 Table — 1DTC migration patterns of anterior and posterior DTCs were analyzed by DIC and florescence optics in L4 larvae or adults. ***P<0.00001; *P<0.01; nsP≥0.01. n = number of gonad arms scored. SE = standard error of the proportion. 2D/V guidance defects result from impairing unc-5 function and reflect the efficacy of the unc-5(RNAi) in the population. 2 ced-10(n3417) is a maternal effect embryonic lethal mutation. Balanced heterozygotes were injected with unc-5(RNAi) and the ced-10(n3417) homozygous progeny (identified as non-dpy) were analyzed for DTC migration defects. 3 ced-10(t1875) is a maternal effect embryonic lethal mutation. Balanced heterozygotes were injected with unc-5(RNAi) and the ced-10(t1875) homozygous progeny identified as GFP(-) were analyzed for DTC migration defects. (DOCX) [file pgen.1005446.s012.docx]

**S5 Table. A/P polarity reversals in *ced-10* mutant alleles treated or not with *unc-5(RNAi)*^1^**

|  | **A/P polarity reversals** | | | | | | **D/V migration failures^2^** | | | | | |
| --- | --- | --- | --- | --- | --- | --- | --- | --- | --- | --- | --- | --- |
|  | **Anterior** | | | **Posterior** | | | **Anterior** | | | **Posterior** | | |
| **Strain** | **%** | **SE** | **n** | **%** | **SE** | **n** | **%** | **SE** | **n** | **%** | **SE** | **n** |
| *ced-10(n3417)^3^* | 16 | 3 | 168 | 23 | 3 | 169 | 0 | 0 | 169 | 0 | 0 | 169 |
| *ced-10(n3417); unc-5(RNAi)* | 9^ns^ | 2 | 150 | 3^***^ | 1 | 149 | 15 | 3 | 186 | 38 | 3 | 188 |
| *unc-5(RNAi)* | 0 | 0 | 87 | 0 | 0 | 87 | 15 | 4 | 87 | 47 | 5 | 87 |
| *ced-10(t1875)^4^* | 26 | 4 | 119 | 19 | 4 | 119 | 0 | 0 | 119 | 1 | 1 | 119 |
| *ced-10(t1875); unc-5(RNAi)* | 18 ^ns^ | 4 | 93 | 6^*^ | 2 | 92 | 14 | 2 | 407 | 26 | 2 | 406 |

^1^DTC migration patterns of anterior and posterior DTCs were analyzed by DIC and florescence optics in L4 larvae or adults. ^***^P<0.00001; ^*^P<0.01; ^ns^P≥0.01.

n = number of gonad arms scored. SE = standard error of the proportion.

^2^D/V guidance defects result from impairing *unc-5* function and reflect the efficacy of the *unc-5(RNAi)* in the population.

^3^*ced-10(n3417)* is a maternal effect embryonic lethal mutation. Balanced heterozygotes were injected with *unc-5(RNAi)* and the *ced-10(n3417)* homozygous progeny (identified as non-dpy) were analyzed for DTC migration defects.

^4^*ced-10(t1875)* is a maternal effect embryonic lethal mutation. Balanced heterozygotes were injected with *unc-5(RNAi)* and the *ced-10(t1875)* homozygous progeny identified as GFP(-) were analyzed for DTC migration defects.
