# Supplementary material for: The Wnt Frizzled Receptor MOM-5 Regulates the UNC-5 Netrin Receptor through Small GTPase-Dependent Signaling to Determine the Polarity of Migrating Cells
Source: PLoS Genet. 2015 Aug 20;11(8):e1005446. doi: 10.1371/journal.pgen.1005446 (PMC4546399; doi:10.1371/journal.pgen.1005446)
Supplement: S6 Table — 1DTC migration patterns of anterior and posterior DTC were analyzed by DIC and florescence optics in L4 larvae or adults. n = number of gonad arms scored. SE = standard error of the proportion. ***P<0.00001; **P<0.001; *P<0.01; nsP≥0.01. 2It should be noted that we refrained from comparing the penetrance of D/V migration failures when unc-5(RNAi) was used. unc-5(RNAi) was delivered by injection; the efficacy of the RNAi treatment can vary between the injected strains. The percent D/V defects in the unc-5(RNAi) set of experiments reflect the efficacy of the RNAi. 3 mig-2(gm103) evIs129/+ were used. Only the emb-9p::gfp positive animals were included in the analysis; these may have been either heterozygous or homozygous for evIs129. (DOCX) [file pgen.1005446.s013.docx]

**S6 Table. A/P polarity reversals or D/V migration defects in *mig-2* null alleles alone or in the background of *unc-5* mutants or *unc-5(RNAi)* as well as DTC migration defects of *mig-2(gm103)* in the presence or absence of *evIs129*^1^**

|  | **A/P polarity reversals** | | | | | | **D/V migration failures^2^** | | | | | |
| --- | --- | --- | --- | --- | --- | --- | --- | --- | --- | --- | --- | --- |
|  | **Anterior** | | | **Posterior** | | | **Anterior** | | | **Posterior** | | |
| **Strain** | **%** | **SE** | **n** | **%** | **SE** | **n** | **%** | **SE** | **n** | **%** | **SE** | **n** |
| *mig-2(mu28)* | 17 | 2 | 456 | 22 | 2 | 456 | 1 | 1 | 456 | 1 | 1 | 456 |
| *unc-5(e53); mig-2(mu28)* | 20^ns^ | 4 | 100 | 10^*^ | 3 | 99 | 38 ^ns^ | 5 | 100 | 75^ns^ | 4 | 99 |
| *unc-5(e53)* | 3 | 1 | 161 | 1 | 1 | 161 | 35 | 4 | 161 | 69 | 4 | 161 |
| *unc-5(ev489); mig-2(mu28)* | 23 ^ns^ | 3 | 226 | 8^***^ | 2 | 226 | 47^***^ | 3 | 226 | 71 ^ns^ | 3 | 226 |
| *unc-5(ev489)* | 3 | 1 | 260 | 0.5 | 0.5 | 260 | 29 | 3 | 260 | 71 | 3 | 260 |
| *unc-5(RNAi); mig-2(mu28)* | 18 ^ns^ | 5 | 71 | 8^*^ | 3 | 71 | 44 | 6 | 71 | 49 | 6 | 71 |
| *unc-5(RNAi)* | 0 | 0 | 87 | 0 | 0 | 87 | 15 | 4 | 87 | 47 | 5 | 87 |
| *mig-2(gm103)* | 20 | 3 | 156 | 24 | 3 | 156 | 20 | 3 | 156 | 20 | 3 | 156 |
| *mig-2(gm103) evIs129[emb-9p::unc-5]^3^* | 9^**^ | 2 | 138 | 9^**^ | 2 | 138 | 6^**^ | 2 | 138 | 7^**^ | 2 | 138 |
| *mig-2(gm103)* 25°C | 27 | 4 | 158 | 34 | 4 | 158 | 15 | 3 | 158 | 39 | 4 | 158 |
| *mig-2(gm103) evIs129[emb-9p::unc-5]^3^* 25°C | 6^***^ | 2 | 108 | 15^**^ | 3 | 108 | 4^*^ | 2 | 108 | 4^***^ | 2 | 158 |

^1^DTC migration patterns of anterior and posterior DTC were analyzed by DIC and florescence optics in L4 larvae or adults. n = number of gonad arms scored. SE = standard error of the proportion. ^***^P<0.00001; ^**^P<0.001; ^*^P<0.01; ^ns^P≥0.01.

^2^It should be noted that we refrained from comparing the penetrance of D/V migration failures when *unc-5(RNAi)* was used. *unc-5(RNAi)* was delivered by injection; the efficacy of the RNAi treatment can vary between the injected strains. The percent D/V defects in the *unc-5(RNAi)* set of experiments reflect the efficacy of the RNAi.

^3^*mig-2(gm103) evIs129/+* were used. Only the *emb-9::gfp* positive animals were included in the analysis, these may have been either heterozygous or homozygous for *evIs129*.
