# Supplementary material for: The Wnt Frizzled Receptor MOM-5 Regulates the UNC-5 Netrin Receptor through Small GTPase-Dependent Signaling to Determine the Polarity of Migrating Cells
Source: PLoS Genet. 2015 Aug 20;11(8):e1005446. doi: 10.1371/journal.pgen.1005446 (PMC4546399; doi:10.1371/journal.pgen.1005446)
Supplement: S7 Table — One way ANOVA column statistics. (DOCX) [file pgen.1005446.s014.docx]

**S7 Table. *mom-5* is transcriptionally activated prior to the onset of phase 3**

**One way ANOVA column statistics**

|  | **phase 1** | **late phase 1** | **phase 2** | **early phase 3** | **mid phase 3** | **late phase 3** |
| --- | --- | --- | --- | --- | --- | --- |
|  |  |  |  |  |  |  |
| **Number of values** | 5 | 6 | 8 | 4 | 15 | 9 |
|  |  |  |  |  |  |  |
| **Minimum** | 105.2 | 267.3 | 293.2 | 1019 | 1137 | 1823 |
| **25% Percentile** | 149.4 | 274.7 | 494 | 1047 | 1423 | 2278 |
| **Median** | 232.5 | 358 | 602.1 | 1141 | 1840 | 2825 |
| **75% Percentile** | 294.1 | 461.9 | 623.1 | 1159 | 2392 | 3219 |
| **Maximum** | 296.9 | 478.3 | 994.8 | 1161 | 2924 | 3522 |
|  |  |  |  |  |  |  |
| **Mean** | 223.9 | 365.9 | 597.9 | 1115 | 1857 | 2785 |
| **Std. Deviation** | 78.95 | 88.31 | 196.4 | 65.76 | 553.1 | 558.6 |
| **Std. Error** | 35.31 | 36.05 | 69.43 | 32.88 | 142.8 | 186.2 |
|  |  |  |  |  |  |  |
| **Lower 95% CI** | 125.9 | 273.2 | 433.7 | 1011 | 1550 | 2355 |
| **Upper 95% CI** | 321.9 | 458.5 | 762.1 | 1220 | 2163 | 3214 |
