# Supplementary material for: The Wnt Frizzled Receptor MOM-5 Regulates the UNC-5 Netrin Receptor through Small GTPase-Dependent Signaling to Determine the Polarity of Migrating Cells
Source: PLoS Genet. 2015 Aug 20;11(8):e1005446. doi: 10.1371/journal.pgen.1005446 (PMC4546399; doi:10.1371/journal.pgen.1005446)
Supplement: S8 Table — 1DTC migration patterns of anterior and posterior DTC were analyzed by DIC in L4 larvae or adults. n = number of gonad arms scored. SE = standard error of the proportion. nsP≥0.01. 2 mom-5(gk812) is a maternal effect embryonic lethal mutation. Balanced heterozygotes were fed with mom-5(RNAi) and the mom-5 mutant homozygous progeny were analyzed for DTC migration defects; the balancer chromosome is marked with gfp hence gk812 homozygotes were identified as non-gfp (S1 Table). (DOCX) [file pgen.1005446.s015.docx]

**S8 Table. *mom-5(gk812)* A/P polarity reversals are not enhanced by *mom-5(RNAi)^1^***

|  | **A/P polarity reversals** | | | | | |
| --- | --- | --- | --- | --- | --- | --- |
|  | **Anterior** | | | **Posterior** | | |
| **Strain** | **%** | **SE** | **n** | **%** | **SE** | **n** |
| *mom-5(RNAi)* | 63 | 5 | 83 | 55 | 5 | 83 |
| *mom-5(gk812)* | 58 | 6 | 80 | 94 | 3 | 80 |
| *mom-5(gk812);mom-5(RNAi)^3^* | 59^ns^ | 7 | 56 | 95 | 3 | 56 |

^1^DTC migration patterns of anterior and posterior DTC were analyzed by DIC in L4 larvae or adults. n = number of gonad arms scored. SE = standard error of the proportion. ^ns^P≥0.01.

^2^*mom-5(gk812)* is a maternal effect embryonic lethal mutation. Balanced heterozygotes were fed with *mom-5(RNAi)* and the *mom-5* mutant homozygous progeny were analyzed for DTC migration defects; the balancer chromosome is marked with gfp hence *gk812* homozygotes were identified as non-*gfp* (S1 Table).
