# Supplementary material for: The Wnt Frizzled Receptor MOM-5 Regulates the UNC-5 Netrin Receptor through Small GTPase-Dependent Signaling to Determine the Polarity of Migrating Cells
Source: PLoS Genet. 2015 Aug 20;11(8):e1005446. doi: 10.1371/journal.pgen.1005446 (PMC4546399; doi:10.1371/journal.pgen.1005446)
Supplement: S9 Table — 1DTC migration patterns of anterior and posterior DTC were analyzed by DIC in L4 larvae or adults. n = number of gonad arms scored. SE = standard error of the proportion. **P<0.001; *P<0.01; nsP≥0.01 2RNAi was introduced by feeding; controls were grown on empty vector feeding bacteria. 3The penetrance of defects in the evIs129 transgenic line was highly temperature sensitive and variable. Care was taken to analyze the respective control for each experiment grown under the same conditions. (DOCX) [file pgen.1005446.s016.docx]

**S9 Table. *evIs129* A/P polarity reversals are enhanced by *mom-5(gk812) and ced-12(k149)^1^***

|  | **A/P polarity reversals** | | | | | |
| --- | --- | --- | --- | --- | --- | --- |
|  | **Anterior** | | | **Posterior** | | |
| **Strain** | **%** | **SE** | **n** | **%** | **SE** | **n** |
| *mom-5(RNAi)* | 63 | 5 | 83 | 55 | 5 | 83 |
| *evIs129[emb-9p::unc-5]^2,3^* | 30 | 4 | 129 | 52 | 4 | 128 |
| *evIs129;mom-5(RNAi)^3^* | 59 | 6 | 78 | 80^**^ | 5 | 78 |
| *evIs129[emb-9p::unc-5]^2,3^* | 33 | 2 | 370 | 40 | 3 | 370 |
| *ced-12(k149);evIs129^3^* | 44^*^ | 2 | 491 | 47^ns^ | 2 | 491 |

^1^DTC migration patterns of anterior and posterior DTC were analyzed by DIC in L4 larvae or adults. n = number of gonad arms scored. SE = standard error of the proportion. ^**^P<0.001; ^*^P<0.01; ^ns^P≥0.01

^2^The penetrance of defects in the *evIs129* transgenic line was highly temperature sensitive and variable. Care was taken to analyze the respective control for each experiment grown under the same conditions.

^3^RNAi was introduced by feeding, controls were grown on empty vector feeding bacteria.
